# Cross metathesis-mediated synthesis of hydroxamic acid derivatives

**DOI:** 10.3762/bjoc.14.285

**Published:** 2018-12-17

**Authors:** Shital Kumar Chattopadhyay, Subhankar Ghosh, Suman Sil

**Affiliations:** 1 Department of Chemistry, University of Kalyani, Kalyani - 741235, West Bengal, India, Fax: +91+33+25828282

**Keywords:** α-amino acid, catalysis, cross metathesis, hydroxamates

## Abstract

An alternative synthesis of α,ß-unsaturated hydroxamates via cross metathesis between a class-I olefin and *N*-benzyloxyacrylamide is reported. The reaction proceeds better in the presence of Grubbs’ second generation catalyst within short time and in good yields (57–85%) with a range of substrates. Subsequent hydrogenation of each of the CM products delivers the title compounds in moderate to very good yield (70–89%). An important demonstration of the protocol is the preparation of the unusual amino acid component of the bioactive cyclic peptide Chap-31.

## Introduction

Cross-metathesis reactions (CM) have rapidly grown [1–3] to be a reliable method for the preparation of functionalized alkenes and derivatives thereof. Intricacies regarding the electronic nature of olefins, their substitution patterns and steric demands are more or less settled through the works of many workers in many reports [4–7]. Yet, a number of new reports describing the CM-mediated synthesis of functionalized alkenes of various kinds continue to appear. For example, cross metathesis with acrylates [8–10], α,ß-unsaturated acid chlorides [11], acrylamides [12–14], vinyl sulfones [15], vinylphosphine oxides [16], vinyl phosphonates [17], enones [18], and nitrile functionalities [19–20] have been shown to yield shorter routes to compounds of interest as well as for green chemical applications [21–22].

**Figure 1:**
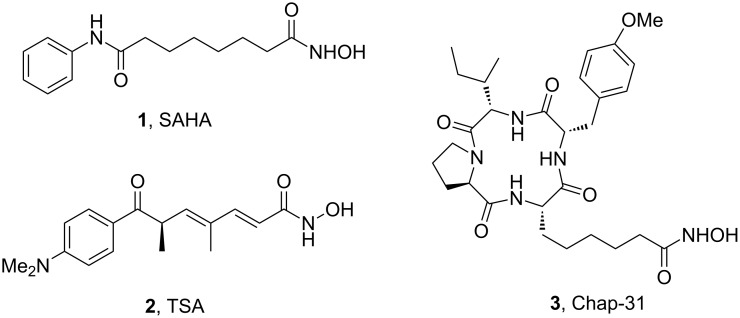
Some bioactive molecules containing hydroxamate functionality.

Hydroxamates belong to a class of valuable biologically relevant compounds of proven record of utility. For example, the hydroxamate SAHA (**1**, Figure 1) [23] and the didehydrohydroxamate TSA (**2**) [24], display useful anticancer properties through inhibition of histone deacetylase enzymes (HDAc) and are used as FDA-approved drugs. Similarly, the cyclic peptide Chap-31 (**3**) [25] with a terminal hydroxamic acid residue has shown promising anticancer activity. Access to such derivatives usually involves the preparation of the corresponding acid and subsequent amide bond formation with hydroxylamines. Although this two-step protocol is widely used, a direct access to α,ß-unsaturated and saturated hydroxamates from cross metathesis of alkenes may prove to be of advantage. In continuation of our earlier studies [26–27] on HDAC inhibitors, we herein report a direct access to α,ß-unsaturated hydroxamates through cross-metathesis reaction.

## Results and Discussion

**Scheme 1:**
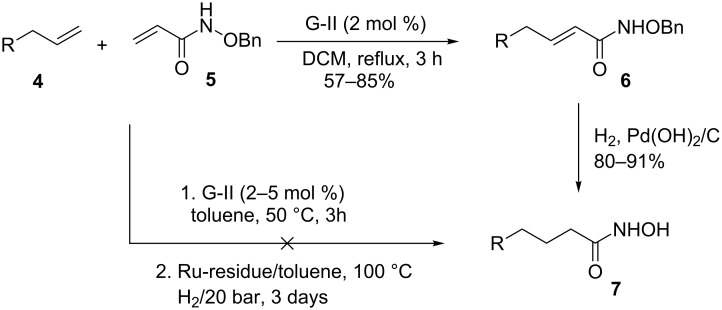
Cross metathesis between a class-I alkene and *N*-benzyloxyacryl amide.

It is known that a CM reaction between a class-I olefin and a class-II olefin proceeds better in the presence of 2nd generation catalysts. Accordingly, CM between 1-decene (**4**, R = C_7_H_15_) and *N*-benzyloxyacrylamide **5** (Scheme 1) was attempted with Grubbs’ second generation catalyst **[**(1,3-bis(2,4,6-trimethylphenyl)-2-imidazolidinylidene)dichloro(phenylmethylene) (tricyclohexylphosphine)ruthenium, G-II]. After some experimentation, it was found that the reaction proceeds quickly in refluxing dichloromethane to provide the CM product **6** (R = C_7_H_15_) in 81% yield. The yield of **6** was improved to 84% when Hoveyda–Grubbs 2nd generation catalyst [1,3-bis-(2,4,6-trimethylphenyl)-2-imidazolidinylidene]dichloro(*o*-isopropoxyphenylmethylene)ruthenium] (HG-II) was used under identical conditions. Hydrogenation of the later in the presence of Pd(OH)_2_/C proceeded uneventfully resulting in the saturation of the double bond as well as concommitant deprotection of the *O*-benzyl group. The one-pot CM-hydrogenation sequence using the same ruthenium catalyst has recently found applications [28–30]. However, similar attempts in our case, i.e., direct conversion of **4 + 5** → **7** proved to be problematic and conversion to the desired product was not observed under the attempted conditions. An intractable mixture of compounds was the result.

Having established the conditions for stepwise CM and hydrogenation reactions, we extended the study to other substrates (Table 1). For example, the yields of the two steps for dodecene forming **6b** and **7b** were more or less similar with those for decene when either of the 2nd-generation catalysts was used. However, analogous reaction with bromobutene **4c** as CM partner proceeded with some compromise in yield with G-II. Moreover, HG-II in this case proved to be less successful. Similarly, the allylbenzene derivatives **4d–f** reacted with more or less similar ease with G-II to produce the corresponding CM products **6d–f**, respectively. Considerable isomerization (1:1 by ^1^H NMR) of the CM-product **6d** to the corresponding styrene derivative was noticed when HG-II was used in place of G-II. **6e** behaved similarly. Reaction with the styrene derivative **4g** resulted in low conversion to the CM product **6g** (57%). Styrene derivatives, belonging to class-I olefins according to Grubbs’ generalizations [31], are indeed known to be a sluggish partner in CM reactions, with homodimerization to stilbene being a recurring problem.

**Table 1:** Hydroxamates prepared.

Entry	Alkene **4**	CM product **6** (% yield)	reduction product **7** (% yield)

1	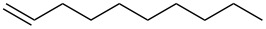 **4a**	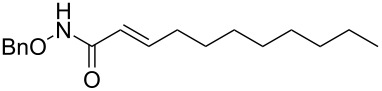 **6a** (81)	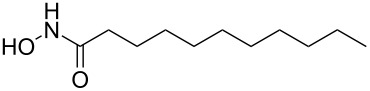 **7a** (85)
2	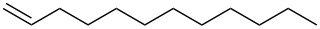 **4b**	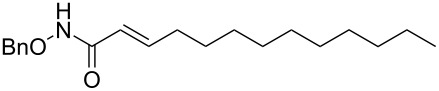 **6b** (85)	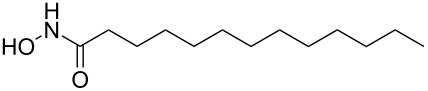 **7b** (83)
3	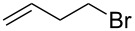 **4c**	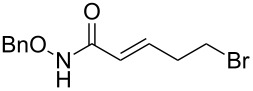 **6c** (72)	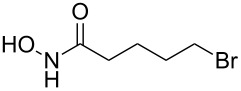 **7c** (70)
4	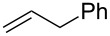 **4d**	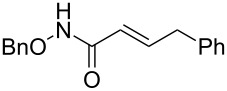 **6d** (77)	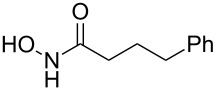 **7d** (89)
5	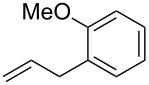 **4e**	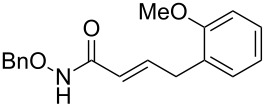 **6e** (72)	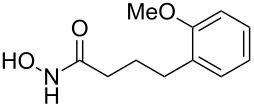 **7e** (85)
6	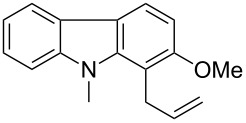 **4f**	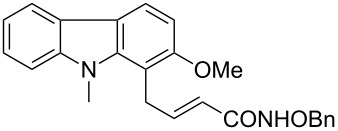 **6f** (78)	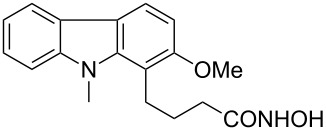 **7f** (80)
7	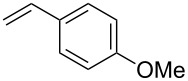 **4g**	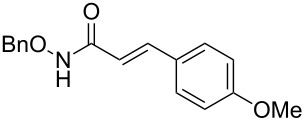 **6g** (57)	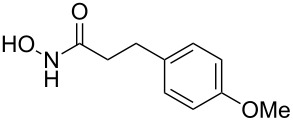 **7g** (83)
8	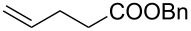 **4h**	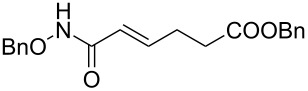 **6h** (79)	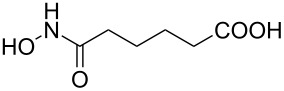 **7h** (81)
9	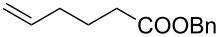 **4i**	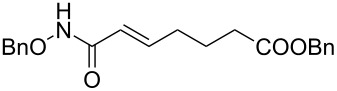 **6i** (73)	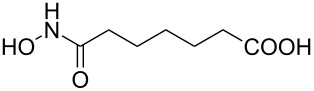 **7i** (78)
10	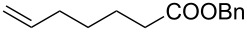 **4j**	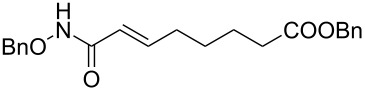 **6j** (70)	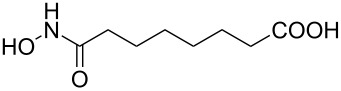 **7j** (75)
11	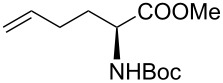 **4k**	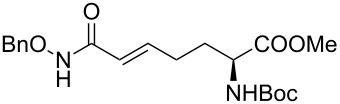 **6k** (78)	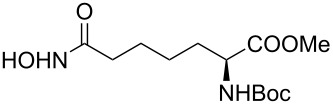 **7k** (86)
12	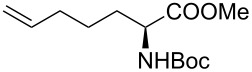 **4l**	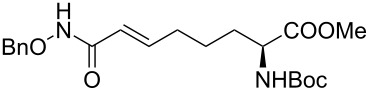 **6l** (78)	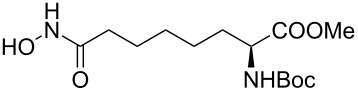 **7l** (84)

Alkenes **4h–j** containing a benzyl ester functionality at two, three and four carbons apart, respectively, participated in the reaction nearly equally well to give the corresponding CM products **6h–j**. Hydrogenation of each of these compounds separately led to the corresponding saturated hydroxamic acid derivatives **7h–j** with concommittant cleavage of the terminal benzyl ester functionality. In an extension to the synthesis of the unusual amino acid component of the important anticancer cyclic peptide compound Chap-31, we attempted the cross-metathesis reaction of *N*-benzyloxyacryl amide **5** with the homoallylglycine derivative **4k** (Table 1, entry 11) and the bis-homoallyl glycine derivative **4l** (Table 1, entry 12) [32], separately. Fortunately, both the reactions proceeded well and the desired amino acid derivatives **7k** and **7l** were obtained in good yields after hydrogenation.

## Conclusion

In conclusion, we have developed a direct access to functionalized hydroxamic acid derivatives using a cross-metathesis reaction between *N*-benzyloxyacylamide and a range of terminal alkenes. The products include hydroxamic acid derivatives with a long alkyl chain, aromatic and heteroaromatic cores, halogen residue, carboxylic acid moiety at the terminal relevant position for drug discovery. Moreover, an alternate preparation of the amino acid component of the important cyclic peptide Chap-31 may encourage the preparation of cyclic peptide based HDAC inhibitors. The developed methodology may hence complement the existing literature on the preparation of such class of compounds and may find applications.

## Experimental

### General procedure for cross metathesis


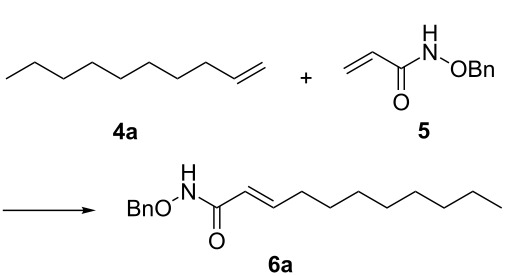


Grubbs’ second generation catalyst G-II (10 mg, 2 mol %), was added to a stirred solution of the olefin **4a** (158 mg, 1.13 mmol), and olefin **5** (100 mg, 0.56 mmol), in anhydrous and degassed CH_2_Cl_2_ (3 mL) at rt and the reaction mixture was heated to reflux for 6 h under argon atmosphere. The reaction mixture was allowed to cool to room temperature and then concentrated in vacuo. The residue was purified by column chromatography on silica gel (hexane/ethyl acetate 60:40) to provide the CM product (*E*)-*N*-benzyloxy)undec-2-enamide (**6a**, 133 mg, 81%) as a colourless viscous liquid.

IR (neat): 3183, 3064, 2926, 2855, 1669, 1683 cm^−1^; ^1^H NMR (400 MHz, DMSO-*d*_6_) δ 11.15 (s, 1H, NH,), 7.38–7.29 (m, 5H, ArH), 6.74–6.67 (m, 1H, C3-H), 5.72 (d, *J* = 15.2 Hz, 1H, C2-H), 4.80 (s, 2H, OCH2-), 2.08 (q, *J* = 6.8 Hz, 2H, C4-H), 1.33 (brs, 3H, CH_2_), 1.12 (s, 12H, CH_2_), 0.81 (t, *J* = 6.8 Hz, 3H, C11-H3); ^13^C NMR (100 MHz, DMSO-*d*_6_) δ 163.4 (CO), 144.3 (C3), 136.4 (ArC), 129.2 (Ar*C*H), 128.7 (Ar*C*H), 121.1 (C2), 77.4 (OCH_2_), 31.8 (C4), 31.7 (C5), 29.3 (CH_2_), 29.1 (CH_2_), 29.0 (CH_2_), 28.2 (CH_2_), 22.6 (CH_2_), 14.3 (C11); HRMS (TOF MS ES^+^) *m/z*: [M + Na]^+^ calcd for C_18_H_27_NNaO_2_, 312.1939; found, 312.1956.

### General procedure for hydrogenation


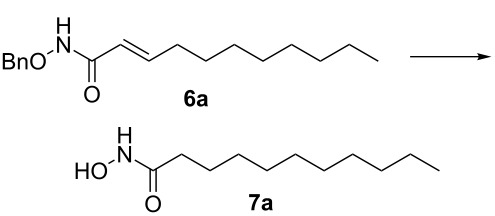


CM product **6a** (50 mg, 0.17 mmol) was taken in a MeOH (3 mL) containing 1 drop of TFA [33]. Then Pd(OH)_2_ (10 mg) was added and the solution was degassed several times. Hydrogen gas was let in and the resulting heterogeneous mixture was vigorously stirred at atmospheric pressure for 2 h. It was filtered through Celite, the filter cake was washed with methanol (5 mL) and the combined filtrate was concentrated in vacuo. The residue was purified by column chromatography on silica gel (CHCl_3_/MeOH 97:3) to provide the product *N*-hydroxyundecanamide **7a** (85%) as a colorless solid.

Mp 85 °C; IR (neat): 3259, 3058, 2956, 1663, 1624 cm^−1^; ^1^H NMR (400 MHz, DMSO-*d*_6_) δ 10.52 (s, 1H, NH), 8.93 (brs, 1H, OH), 1.92 (t, *J* = 7.2 Hz, 2H, C2-H), 1.44 (m, 2H, C3-H), 1.19 (s, 14H, 7× CH_2_), 0.81 (t, *J* = 6.8 Hz, 3H, C11-H); ^13^C NMR (100 MHz, DMSO-*d*_6_) δ 170.4 (CO), 32.6 (C2), 31.6 (C3), 29.3 (CH_2_), 29.3 (CH_2_), 29.1 (CH_2_), 28.9 (CH_2_), 25.5 (CH_2_), 22.5 (CH_2_), 14.3 (C11); HRMS (TOF MS ES^+^) *m/z*: [M + Na]^+^ calcd for C_11_H_23_NNaO_2_, 224.1626; found, 224.1638.

## Supporting Information

File 1 Analytical data of all new compounds as well as copies of their ^1^H and ^13^C NMR spectra.
