# Supplementary material for: Cross metathesis-mediated synthesis of hydroxamic acid derivatives
Source: Beilstein J Org Chem. 2018 Dec 17;14:3070–5. doi: 10.3762/bjoc.14.285 (PMC6317413; doi:10.3762/bjoc.14.285)
Supplement: File 1 — Analytical data of all new compounds as well as copies of their 1H and 13C NMR spectra. [file Beilstein_J_Org_Chem-14-3070-s001.pdf]

BEILSTEIN JOURNAL OF ORGANIC CHEMISTRY

Supporting Information

for

Cross metathesis-mediated synthesis of hydroxamic acid derivatives

Shital Kumar Chattopadhyay, Subhankar Ghosh and Suman Sil

Beilstein J. Org. Chem. **2018,** *14,* 3070–3075. doi:10.3762/bjoc.14.285

Analytical data of all new compounds as well as copies of their ^{1}H and ^{13}C NMR spectra

License and Terms: This is a supporting information file under the terms of the Creative Commons Attribution License (http://creativecommons.org/licenses/by/4.0). Please note that the reuse, redistribution and reproduction in particular requires that the authors and source are credited.
The license is subject to the *Beilstein Journal of Organic Chemistry* terms and conditions: (https://www.beilstein-journals.org/bjoc)

Content **page**

Characterization data for compounds **6a–l** and **7a–l** S2–18

Copies of ^{1}H and ^{13}C NMR spectra of the reported compounds.............................. S19–60

General information

General: Column chromatography was performed on silica gel, Merck grade 230–400 mesh and neutral alumina. Reactions were monitored by thin-layer chromatography; TLC plates were visualized with UV light, in an iodine chamber, or with vaniline solution, unless noted otherwise. Melting points were recorded in open capillaries and are uncorrected. IR spectra were recorded using KBr disks, chloroform solution or neat. Chemical shifts (δ) are given from TMS (0 ppm) as internal standard for ^{1}H NMR and $^{13}CDCl_3$ (77.0 ppm) for ^{13}C NMR. The following abbreviations were used to explain the multiplicities: s = singlet, d = doublet, t = triplet, q = quartet, m = multiplet, dd = double doublet, ddd = doublet of double doublet, dt = doublet of triplet, br = broad, etc. HRMS data were recorded on a Waters XEVO G2 QTOF instrument purchased through DST-PURSE Grant.

Dichloromethane, dimethyl sulfoxide were distilled over calcium hydride under an inert atmosphere. THF, toluene, benzene and ether were freshly distilled under argon from a purple solution of sodium benzophenone ketyl. Unless stated otherwise, all reagents were purchased from commercial sources and used without additional purification.

(*E*)-*N*-(Benzyloxy)tridec-2-enamide (6b)

Eluent: hexane:ethyl acetate (60:40)

Yield: 85%. Colorless viscous liquid.

IR (neat): 3429, 3200, 2924, 2853, 1666, 1634 cm^{-1}.

^{1}H NMR (400 MHz, DMSO-d_6): δ 11.11 (1H, s, NH), 7.62-7.40 (5H, s, ArH), 6.79-6.71 (1H, m, C3-H), 5.76 (1H, d, *J* = 14.8, C2-H), 4.86 (2H, s, OCH2), 2.17 (2H, m, C4-H), 1.42 (3H, brs, CH2), 1.29 (13H, s, CH2), 0.90 (3H, t, *J* = 3.6Hz, C13-H3).

^{13}C NMR (100 MHz, DMSO-d_6): δ 163.3 (CO), 144.2 (C3), 136.5 (ArC), 129.2 (ArCH), 128.8(ArCH), 128.7(ArCH), 121.3(C2), 77.4 (OCH2), 31.8(C4), 31.7(C5), 29.5 (CH2), 29.3(CH2), 29.2(CH2), 29,0(CH2), 28.2(CH2), 22.6(CH2), 14.4 (C13).

HRMS (TOF MS ES+): *m/z* [M + Na] $^{+}$ calcd. for $C_{20}H_{31}NNaO_2$ 340.2252; found 340.2243

(*E*)-*N*-(Benzyloxy)-5-bromopent-2-enamide (6c):

Eluent: hexane:ethylacetate (65:35).

Yield: 72%. Colorless viscous liquid.

IR (neat): 3201, 3031, 2963, 2887, 1958, 1667, 1635 cm^{-1}.

NMR (400 MHz, DMSO-d_6): δ 11.27 (1H, s, NH), 7.45-7.39 (5H, m, ArH), 6.77-6.69 (1H, m, C3-H), 5.88 (1H, d, *J* = 15.2Hz, C2-H), 4.89 (2H, s, OCH2), 3.66 (2H, t, *J* = 6.8 Hz, CH2Br), 3.46 (s, H_2O from DMSO-d6), 2.77 (2H, q, *J* = 6.4Hz, C4-H_2).

^{13}C NMR (100 MHz, DMSO-d_6): δ 162.8(CO), 140.9(C3), 136.4(ArC), 129.2(ArCH),128.8(ArCH), 128.8(ArCH), 123.4(C2), 77.4 (OCH2) , 34.9 (C5) , 33.0 (C4).

Anal calcd for $C_{12}H_{14}BrNO_2$ C, 50.72; H, 4.97; N, 4.93; observed, C, 50.60; H, 4.78; N, 4.71.

(*E*)-*N*-(Benzyloxy)-4-phenylbut-2-enamide (6d):

Eluent: hexane:ethylacetate (60:40).

Yield: 77%. Colorless viscous liquid.

IR (neat): 3124, 3028, 2930, 1670, 1642 cm^{-1}.

^1H NMR (400 MHz, DMSO-d_6): δ 11.21 (1H, s, NH), 7.42-7.36 (8H, m, ArH), 7.30-7.24 (2H, m, ArH), 6.88 (1H, m, C3-H), 5.74 (1H, d, *J* = 15.6Hz, C2-H), 4.85 (2H, s, OCH2), 3.53 (2H, d, *J* = 6.8 Hz, ArCH2).

^{13}C NMR (100 MHz, DMSO-d_6): δ 163.1 (CO), 143.1(C3), 138.9 (ArC), 136.5 (ArC), 129.2 (ArCH), 129.0 (ArCH), 128.8(ArCH), 126.8(ArCH), 122.0 (C2), 77.4 (OCH2) , 37.9 (C4).

HRMS (TOF MS ES+): *m/z* $[M + Na]^+$ calcd. for $C_{17}H_{17}NNaO_2$ 290.1157; found 290.1154

(*E*)-*N*-(Benzyloxy)-4-(2-methoxyphenyl)but-2-enamide (6e):

Eluent: hexane:ethyl acetate (50:50)

Yield: 72%. Colorless viscous liquid.

IR (neat): 3153, 3028, 2960, 2838, 1805, 1667, 1634 cm^{-1}.

^1H NMR (400 MHz, DMSO-d_6): δ 11.13 (1H, s, NH), 7.37 (5H, brs, ArH), 7.23 (1H, t, *J* =7.6Hz, ArH), 7.11 (1H, d, *J* = 7.2Hz, ArH), 6.98 (1H, d, *J* = 8.4Hz, ArH), 6.90-6.79 (2H, m, ArH+C3-H), 5.65 (1H, d, *J* = 15.6Hz, C2-H), 4.80 (2H, s, OCH2), 3.78 (3H, s, OCH3), 3.41 (H_2O from DMSO-d6).

^{13}C NMR (100 MHz, DMSO-d_6): δ 163.2 (CO), 157.3 (O-ArC), 142.5(C3), 136.5(ArC), 130.4(ArC), 129.2(ArCH), 128.8 (ArCH, two signals), 128.5(ArCH), 126.7(ArCH), 121.6 (ArCH), 120.9 (C2), 111.3(ArCH), 77.4 (OCH2), 55.8(OCH3), 32.6 (C4).

HRMS (TOF MS ES+): *m/z* [M + Na] $^{+}$ calcd. for $C_{18}H_{19}NNaO_3$ 320.1263; found 320.1269.

(*E*)-*N*-(Benzyloxy)-4-(2-methoxy-9-methyl-9*H*-carbazol-1-yl)but-2-enamide (6f):

Eluent: hexane:ethylacetate (50:50).

Yield: 78%. Light yellow solid.

M.p-160-161°C.

IR (neat): 3232, 2997, 2933, 1661, 1634 cm^{-1}.

^{1}H NMR (400 MHz, DMSO-d_6): δ 11.01 (1H, s, NH), 8.01 (2H, d, *J* = 7.6Hz, ArH), 7.48 (1H, d, *J* = 8Hz, ArH), 7.38-7.31 (6H, m, ArH), 7.17-7.07 (2H, m, ArH+C3-H), 6.98 (1H, d, *J* = 8Hz, ArH), 5.41 (1H, d, *J* = 15.6 Hz, C2-H), 4.73 (2H, s, OCH2), 4.02 (2H, brs, ArCH2), 3.91 (3H, s, OMe), 3.85 (3H, s, NMe).

^{13}C NMR (100 MHz, DMSO-d_6): δ 163.7 (CO), 156.8 (OArC), 145.0 (ArC), 142.1 (C3), 140.4 (ArC), 135.8 (ArC), 129.2 (ArCH), 128.9 (ArCH), 128.8 (ArCH), 125.4 (ArCH), 122.5(ArC), 120.9 (ArC), 119.7 (ArCH), 119.6 (ArCH), 119.4 (ArCH), 118.1, 109.3(ArCH), 108.2 (ArC), 104.9(ArCH), 77.4 (OCH2), 57.0 (OCH3), 32.0 (NCH3), 27.0 (C4).

HRMS (TOF MS ES+): *m/z* [M + Na] $^{+}$ calcd for $C_{25}H_{24}N_2NaO_3$ 423.1685; found 423.1678

(*E*)-*N*-(Benzyloxy)-3-(4-methoxyphenyl)acrylamide (6g):

Eluent: hexane: ethyl acetate (50:50).

Yield: 57%. Colourless viscous liquid.

IR (neat): 3135, 2957, 2927, 2854, 2046, 1882, 1665, 1647 cm^{-1}.

^{1}H NMR (400 MHz, DMSO-d_6): δ 11.25 (1H, s, NH), 7.58-7.39 (7H, m, ArH), 7.02 (2H, d, *J* = 8.8 Hz, Ar H), 6.34 (1H, d, *J* = 15.6Hz, C3-H), 4.91 (2H, s, OCH2), 3.82 (3H, s, OCH3), 3.43 (H2O from DMSO-d6).

^{13}C NMR (100 MHz, DMSO-d_6): δ 163.9 (CO), 161.0 (OArC), 139.8 (C3), 136.5 (ArC), 129.8(ArCH), 129.3 (ArCH), 128.8 (ArCH, two signals), 127.6 (ArCH), 116.4 (C2), 114.8(ArCH), 77.5 (OCH2), 55.7(OCH3).

HRMS (TOF MS ES+): *m/z* [M + Na] $^{+}$ calcd. for $C_{17}H_{17}NNaO_3$ 306.1106; found 306.1115

(*E*)-Benzyl 6-(benzyloxyamino)-6-oxohex-4-enoate (6h):

Eluent: hexane:ethylacetate (60:40).

Yield: 79%. Colorless viscous liquid.

IR (neat): 3189, 3064, 3032, 2960, 1735, 1671, 1638 cm^{-1}.

^{1}H NMR (400 MHz, DMSO-d_6): δ 11.09 (1H, s, NH), 7.34-7.24 (10H, m, ArH), 6.65 (1H, dt, *J* = 15.6, 6.8Hz, C4-H), 5.69 (1H, d, *J* = 15.6Hz, C5-H), 5.03 (2H, s, CO_2CH_2Ph), 4.75 (2H, s, OCH_2Ph), 2.48-2.43 (3H, m, C2-H+residual DMSO), 2.35 (2H, q, *J* = 6.8Hz, C3-H).

^{13}C NMR (100 MHz, DMSO-d_6): δ 172.4 ($\underline{C}O_2Bn$), 163.0 ($\underline{C}ONHOBn$), 142.4 (C4), 136.6 (ArC), 136.5 (ArC), 129.2 (ArCH), 128.9 (ArCH), 128.8 (ArCH), 128.7 (ArCH), 128.5 (ArCH), 128.4 (ArCH), 122.0 (C5), 77.4(NHO-$\underline{C}H_2Ph$), 66.0 (COO-$\underline{C}H_2Ph$), 32.5(C2) , 27.2 (C3).

HRMS (TOF MS ES+): *m/z* [M + Na] $^{+}$ calcd. for $C_{20}H_{21}NNaO_4$ 362.1368; found 362.1360

(*E*)-Benzyl 7-(benzyloxyamino)-7-oxohept-5-enoate (6i):

Eluent: hexane:ethyl acetate (60:40).

Yield: 73%. Colorless viscous liquid.

IR (neat): 3381, 3196, 3032, 2928, 1732, 1670, 1641 cm^{-1}.

^{1}H NMR (400 MHz, DMSO-d_6): δ 11.13 (1H, s, NH), 7.39-7.26 (10H, m, ArH), 6.73-6.08 (1H, m, C5-H), 5.74 (1H, d, *J* = 15.6Hz, C6-H), 5.09 (2H, s, $CO_2\underline{CH_2}Ph$), 4.82 (2H, s, $CONHO\underline{CH_2}Ph$), 2.37 (2H, t, *J* = 7.6Hz, C2-H), 2.16 (2H, q, *J* = 6.4Hz, C4-H), 1.71-1.65 (2H, m, C3-H).

^{13}C NMR (100 MHz, DMSO-d_6): δ 172.9 (C1), 163.2 (C7), 143.3 (C5), 136.7(ArC), 136.5(ArC), 129.2 (ArCH), 128.9 (ArCH), 128.8 (ArCH), 128.7 (ArCH), 128.5 (ArCH), 128.4 (ArCH), 121.8 (C6), 77.4($CONHO\underline{C}H_2Ph$) , 65.9 ($CO_2\underline{C}H_2Ph$), 33.3(C2), 31.1(C4), 23.5(C3).

HRMS (TOF MS ES+): *m/z* $[M + Na]^+$ calcd. for $C_{21}H_{23}NNaO_4$ 376.1525; found 376.1519.

(*E*)-Benzyl 8-(benzyloxyamino)-8-oxooct-6-enoate (6j):

Eluent: hexane:ethylacetate (60:40).

Yield: 70%. Colorless viscous liquid.

IR (neat): 3186, 3032, 2933, 1732, 1667, 1533 cm^{-1}.

^{1}H NMR (400 MHz, DMSO-d_6): δ 11.16 (1H, s, NH), 7.43-7.35 (10H, m, ArH), 6.77-6.69 (1H, m, C6-H), 5.76 (1H, d, *J*=15.2Hz, C7-H), 5.12 (2H, s, $CO_2\underline{CH_2}Ph$), 4.86 (2H, s,

CONHOC̲H̲$_2$Ph), 2.42 (2H, t, *J* = 7.6Hz, C2-H), 2.17 (2H, q, *J* = 6Hz, C5-H), 1.62-1.54 (2H, m, C4-H), 1.46-1.39 (2H, m, C3-H).

^{13}C NMR (100 MHz, DMSO-d$_6$): δ 173.1 (C1), 163.3 (C8), 143.9 (C6), 136.7(ArC), 136.5(ArC), 129.2 (ArCH), 128.9 (ArCH), 128.8 (ArCH), 128.7(ArCH), 128.4(ArCH), 128.4(ArCH), 121.4 (C7), 77.4 (CONHOC̲H$_2$Ph), 65.8 (CO$_2$C̲H$_2$Ph), 33.7(C2), 31.4(C5), 27.5(C3), 24.4(C4).

HRMS (TOF MS ES+): *m/z* [M + Na]$^+$ calcd. for $C_{22}H_{25}NNaO_4$ 390.1681; found 390.1684

(*S*,*E*)-Methyl 7-(benzyloxyamino)-2-((*tert*-butoxycarbonyl)amino)-7-oxohept-5-enoate (6k) :

Eluent: hexane : ethyl acetate (60:40).

Yield: 78%. Colorless viscous liquid.

$[\alpha]_D^{25}$= +20.4 (c 1.1, $CHCl_3$).

IR (neat): 3366, 3237, 2977, 2953, 2869, 1726, 1714, 1654 cm^{-1}.

^{1}H NMR (400 MHz, DMSO-d6): δ 11.23 (1H, brs, NH), 7.45-7.41 (6H, m, ArH+NHBoc), 6.70 (1H, m,C5-H), 5.82 (1H, d, *J* = 15.6Hz, C6-H), 4.87 (2H, s, OCH̲$_2$Ph), 4.14 (1H, m, C2-H), 3.68 (3H, s, CO$_2$Me), 3.42 (H_2O from DMSO-d6), 2.56-2.47 (4H, m, merged,C3-H+C4-H) 1.44 (9H,s, O-CMe$_3$).

^{13}C NMR (100 MHz, $CDCl_3$): δ 172.6 (C1), 162.8 (C7), 155.9 (C̲OOCMe$_3$), 139.5 (C5), 136.4 (ArC), 129.2 (ArCH), 128.7 (ArCH, two signals), 123.8 (C6), 78.9 (OC̲Me$_3$), 77.4 (CONHOC̲H$_2$Ph), 53.3 (CO$_2$M̲e̲), 52.3(C2), 33.6(C4), 28.3(OCM̲e̲$_3$ + C-3, two signals).

HRMS (TOF MS ES+): *m/z* [M + Na]$^+$ calcd. for $C_{20}H_{28}N_2NaO_6$ 415.1845; found 415.1857.

(*S*,*E*)-Methyl 8-(benzyloxyamino)-2-((*tert*-butoxycarbonyl)amino)-8-oxooct-6-enoate (6l):

Eluent-hexane : ethyl acetate (60:40).

Yield: 75%. Colorless viscous liquid.

$[\alpha]_D^{25}$= +11.0 (c 0.2, $CHCl_3$).

IR (neat): 3447, 3267, 2977, 2753, 2869, 1757, 1714, 1644 cm^{-1}.

^{1}H NMR (400 MHz,$CDCl_3$): δ 8.76 (1H, brs, N<u>H</u>OBn), 7.31-7.28 (5H, m, ArH), 6.85-6.78 (1H, m, C6-H), 5.63 (1H, brs, N<u>H</u>Boc), 5.05 (1H, m, C7-H), 4.83 (2H, s, OC$\underline{H}_2$Ph), 4.23-4.18 (1H, m, C2-H), 3.65 (3H, s, $CO_2\underline{Me}$), 2.12 (2H, s, C5-H), 1.70 (1H, brs, C3-$\underline{H}_a H_b$), 1.56-1.49 (1H, m, C3-$H_a\underline{H}_b$), 1.46-1.39 (2H, m, C4-H), 1.36 (9H, s, $CO_2C\underline{Me}_3$).

^{13}C NMR (100 MHz, $CDCl_3$): δ 173.2 (C1), 164.6 (C8), 155.4($\underline{C}OOCMe_3$), 145.2 (C6), 135.5(ArC), 129.2(ArCH), 128.8(ArCH), 128.6(ArCH), 120.2 (C7), 80.0 ($O\underline{C}Me_3$), 78.3($CONHO\underline{C}H_2Ph$), 53.1($CO_2\underline{Me}$), 52.3(C2), 32.2 (C5), 31.6 (C3), 28.3 ($OC\underline{Me}_3$), 23.8 (C4).

HRMS (TOF MS ES+): *m/z* [M + Na] $^+$ calcd. for $C_{21}H_{30}N_2NaO_6$ 429.2002; found 429.2009

HRMS (TOF MS ES+): *m/z* [M + Na] $^+$ calcd. for $C_{21}H_{23}NNaO_4$ 376.1525; found 376.1519.

(*E*)-Benzyl 8-(benzyloxyamino)-8-oxooct-6-enoate (6j):

Eluent: hexane:ethylacetate (60:40).

Yield: 70%. Colorless viscous liquid.

IR (neat): 3186, 3032, 2933, 1732, 1667, 1533 cm^{-1}.

^{1}H NMR (400 MHz, DMSO-d_6): δ 11.16 (1H, s, NH), 7.43-7.35 (10H, m, ArH), 6.77-6.69 (1H, m, C6-H), 5.76 (1H, d, *J*=15.2Hz, C7-H), 5.12 (2H, s, $CO_2\underline{CH_2}Ph$), 4.86 (2H, s, CONHO$\underline{CH_2}$Ph), 2.42 (2H, t, *J* = 7.6Hz, C2-H), 2.17 (2H, q, *J* = 6Hz, C5-H), 1.62-1.54 (2H, m, C4-H), 1.46-1.39 (2H, m, C3-H).

^{13}C NMR (100 MHz, DMSO-d_6): δ 173.1 (C1), 163.3 (C8), 143.9 (C6), 136.7(ArC), 136.5(ArC), 129.2 (ArCH), 128.9 (ArCH), 128.8 (ArCH), 128.7(ArCH), 128.4(ArCH), 128.4(ArCH), 121.4 (C7), 77.4 (CONHO$\underline{C}H_2$Ph), 65.8 ($CO_2\underline{C}H_2Ph$), 33.7(C2), 31.4(C5), 27.5(C3), 24.4(C4).

HRMS (TOF MS ES+): *m/z* $[M + Na]^+$ calcd. for $C_{22}H_{25}NNaO_4$ 390.1681; found 390.1684

(*S*,*E*)-Methyl 7-(benzyloxyamino)-2-((*tert*-butoxycarbonyl)amino)-7-oxohept-5-enoate (6k):

Eluent: hexane : ethyl acetate (60:40).

Yield: 78%. Colorless viscous liquid.

$[\alpha]_D^{25}$= +20.4 (c 1.1, $CHCl_3$).

IR (neat): 3366, 3237, 2977, 2953, 2869, 1726, 1714, 1654 cm^{-1}.

^{1}H NMR (400 MHz, DMSO-d6): δ 11.23 (1H, brs, NH), 7.45-7.41 (6H, m, ArH+NHBoc), 6.70 (1H, m,C5-H), 5.82 (1H, d, *J* = 15.6Hz, C6-H), 4.87 (2H, s, OC$\underline{H_2}$Ph), 4.14 (1H, m, C2-H), 3.68 (3H, s, CO_2Me), 3.42 (H_2O from DMSO-d6), 2.56-2.47 (4H, m, merged,C3-H+C4-H) 1.44 (9H,s, O-CMe_3).

^{13}C NMR (100 MHz, DMSO-d6): δ 172.6 (C1), 162.8 (C7), 155.9 ($\underline{C}OOCMe_3$), 139.5 (C5), 136.4 (ArC), 129.2 (ArCH), 128.7 (ArCH, two signals), 123.8 (C6), 78.9 ($O\underline{C}Me_3$), 77.4 ($CONHO\underline{C}H_2Ph$), 53.3 ($CO_2\underline{Me}$), 52.3(C2), 33.6(C4), 28.3($OC\underline{Me_3}$ + C-3, two signals).

HRMS (TOF MS ES+): *m/z* $[M + Na]^+$ calcd. for $C_{20}H_{28}N_2NaO_6$ 415.1845; found 415.1857.

(*S*,*E*)-Methyl 8-(benzyloxyamino)-2-((*tert*-butoxycarbonyl)amino)-8-oxooct-6-enoate (6l):

Eluent- hexane : ethyl acetate (60:40).

Yield: 75%. Colorless viscous liquid.

$[\alpha]_D^{25}$= +11.0 (c 0.2, $CHCl_3$).

IR (neat): 3447, 3267, 2977, 2753, 2869, 1757, 1714, 1644 cm^{-1}.

^{1}H NMR (400 MHz,$CDCl_3$): δ 8.76 (1H, brs, $N\underline{H}OBn$), 7.31-7.28 (5H, m, ArH), 6.85-6.78 (1H, m, C6-H), 5.63 (1H, brs, $N\underline{H}Boc$), 5.05 (1H, m, C7-H), 4.83 (2H, s, $OC\underline{H_2}Ph$), 4.23-4.18 (1H, m, C2-H), 3.65 (3H, s, $CO_2\underline{Me}$), 2.12 (2H, s, C5-H), 1.70 (1H, brs, C3-$\underline{H_a}H_b$), 1.56-1.49 (1H, m, C3-$H_a\underline{H_b}$), 1.46-1.39 (2H, m, C4-H), 1.36 (9H, s, $CO_2C\underline{Me_3}$).

^{13}C NMR (100 MHz, $CDCl_3$): δ 173.2 (C1), 164.6 (C8), 155.4($\underline{C}OOCMe_3$), 145.2 (C6), 135.5(ArC), 129.2(ArCH), 128.8(ArCH), 128.6(ArCH), 120.2 (C7), 80.0 ($O\underline{C}Me_3$), 78.3($CONHO\underline{C}H_2Ph$), 53.1($CO_2\underline{Me}$), 52.3(C2), 32.2 (C5), 31.6 (C3), 28.3 ($OC\underline{Me_3}$), 23.8 (C4).

HRMS (TOF MS ES+): *m/z* $[M + Na]^+$ calcd. for $C_{21}H_{30}N_2NaO_6$ 429.2002; found 429.2009

***N*-Hydroxytridecanamide (7b):**

Eluent: $CHCl_3$: MeOH (97:3).

Yield: 83%. Colorless solid.

M.p 94°C.

IR (neat): 3259, 3056, 2914, 2847, 1662, 1625 cm^{-1}.

^{1}H NMR (400 MHz, DMSO-d_6): δ 10.41 (1H, s, NH), 8.74 (1H, s, OH), 3.97 (H_2O from DMSO-d6), 2.57 (residual DMSO), 1.98 (2H, t, *J* = 7.2Hz, C2-H), 1.53-1.50 (2H, m, C3-H), 1.29 (18H, brs,C4-C12 9 x CH2) , 0.91 (3H, t, *J* = 7.2Hz, C13-H).

^{13}C NMR (100 MHz, DMSO-d_6): δ 169.7(C1), 32.7(C2), 31.7(CH2), 29.5(2 x CH2), 29.4 (2x CH2), 29.2 (CH2), 29.0 (2 x CH2), 25.6(CH2), 22.5(CH2), 14.4(C13).

HRMS (TOF MS ES+): *m/z* $[M + Na]^+$ calcd. for $C_{13}H_{27}NNaO_2$ 252.1939 found 252.1926.

5-Bromo-*N*-hydroxypentanamide (7c):

Eluent: $CHCl_3$: MeOH (97:3).

Yield: 70%. Colorless viscous liquid.

IR (neat): 3359, 3158, 2856, 1667, 1624 cm^{-1}

^{1}H NMR (400 MHz, in $CDCl_3$+ DMSO-d_6): δ 10.45 (1H, s, NH), 3.55 (H_2O from DMSO-d6 + merged 2H, C5-H), 2.57 (residual DMSO), 2.04 (2H, t, *J* = 7.2Hz, C2-H), 1.84 (2H, t, *J* = 7.2Hz, C4-H), 1.77-1.66 (2H, m, C3-H).

^{13}C NMR (100 MHz, in $CDCl_3$+ DMSO-d_6): δ 168.7(C1), 34.1(C5), 31.6(C4), 31.2(C2), 23.7(C3).

Anal calcd. for $C_5H_{10}BrNO_2$ C, 30.63; H, 5.14; N, 7.14. found: C, 30.86; H, 5.40; N, 6.87

***N*-Hydroxy-4-phenylbutanamide (7d):**

Eluent: $CHCl_3$:MeOH (99:1).

Yield: 89%. Colorless solid.

M.p-52°C.

IR (neat): 3413, 3062, 3026, 2925, 2871, 1705, 1692, 1653 cm^{-1}.

^{1}H NMR (400 MHz, DMSO-d_6): δ 7.35-7.32 (2H, m, ArH), 7.25-7.21 (3H, m), 3.42 (H_2O from DMSO-d6), 2.65-2.59 (2H, m, C4-H), 2.26 (2H, t, *J* = 7.6Hz, C2-H), 1.84 (2H, quin, *J* = 7.6Hz, C3-H).

^{13}C NMR (100 MHz, DMSO-d_6): δ 169.4 (C1), 142.1 (ArC), 128.8 (ArCH, two signals), 126.3 (ArCH), 35.0 (C4), 32.2 (C2), 27.4 (C3).

HRMS (TOF MS ES+): *m/z* $[M + Na]^+$ calcd. for $C_{10}H_{13}NNaO_2$ 202.0844; found 202.0842.

***N*-Hydroxy-4-(2-methoxyphenyl)butanamide (7e):**

Eluent: $CHCl_3$:MeOH (97:3).

Yield: 85%. Colorless viscous liquid.

IR (neat): 3218, 3003, 2925, 1783, 1646, 1601cm^{-1}.

^{1}H NMR (400 MHz, $CDCl_3$+ DMSO-d_6): δ 10.39 (1H, s, NH), 8.71 (1H, s, OH), 7.16-7.083 (2H, m, ArH), 6.92-6.84 (3H, m, ArH), 3.76 (3H, s, OMe), 3.58 (H_2O from DMSO-d6), 2.52 (C4-H+residual DMSO), 1.97 (2H, t, *J* = 6.8Hz, C2-H), 1.76-1.72 (2H, m, C3-H).

^{13}H NMR (100 MHz, in $CDCl_3$+ DMSO-d_6): δ 169.7 (C1), 157.4 (Ar$\underline{C}$-OCH_3), 129.9 (ArCH), 127.4 (ArC), 120.5 (ArCH), 110.7 (ArCH), 55.4 (O$\underline{C}H_3$), 32.6 (C4), 29.6 (C2), 25.9 (C3).

HRMS (TOF MS ES+): *m/z* $[M + Na]^+$ calcd. for $C_{11}H_{15}NNaO_3$ 232.0949; found 232.0945.

_N_-Hydroxy-4-(2-methoxy-9-methyl-9_H_-carbazol-1-yl)butanamide (7f):

Eluent: $CHCl_3$:MeOH (97:3).

Yield: 80%. Light yellow solid.

M.p-142-143 °C.

IR (neat): 3267, 3028, 2965, 2933, 1783, 1661, 1634 cm^{-1}.

^{1}H NMR (400 MHz, DMSO-d_6): δ 10.39 (1H, s, NH), 8.70 (1H, s, OH), 7.94 (1H, d, J = 7.6 Hz, ArH), 7.88 (1H, d, J = 8.4 Hz, ArH), 7. 46 (1H, d, J = 8.0 Hz, ArH), 7. 30 (1H, t, J = 7.6 Hz, ArH), 7.07 (1H, t, J = 7.6 Hz, ArH), 6.87 (1H, d, J = 8.8 Hz, ArH), 3.94 (3H, s,OCH_3), 3.81 (3H, s, NCH_3), 3.39 (H_2O from DMSO-d6), 3.01(2H, t, J = 8.0 Hz, C4-H), 2.44 (residual DMSO), 2.06 (2H, t, J = 7.2 Hz, C2-H), 1.79-1.71 (2H, m, C3-H).

^{13}C NMR (100 MHz, DMSO-d_6): δ 169.5(C1), 156.8 (Ar$\underline{C}$-OCH_3), 142.4 (ArCH, 140.2(ArCH, 125.1(ArCH),122.7(ArC),119.4(ArCH),119.3(ArCH),118.7(ArC),118.2(ArC),112.8(ArC),109.4(ArCH), 104.8(ArCH), 56.9(O$\underline{C}H_3$), 32.8(N$\underline{C}H_3$), 32.5(C2), 27.5 (C3), 24.0 (C4).

HRMS (TOF MS ES+): *m/z* $[M + Na]^+$ calcd. for $C_{18}H_{20}N_2NaO_3$ 335.1372; found 335.1374.

_N_-Hydroxy-3-(4-methoxyphenyl)propanamide (7g):

Eluent: $CHCl_3$:MeOH (99:1).

Yield: 83%. Colorless viscous liquid.

IR (neat): 3278, 3033, 2958, 2925, 2853, 2044, 1885, 1859, 1791, 1663, 1612 cm^{-1}.

^{1}H NMR (400 MHz, DMSO-d_6): δ 10.37 (1H, s, NH), 8.72 (1H, s, OH), 7.18-7.09 (2H, m, ArH), 6.82 (2H, d, *J* = 8Hz, ArH), 3.70 (3H, s, OCH_3), 3.41 (H_2O from DMSO-d6), 2.73 (2H, t, *J* = 7.6Hz, C3-H), 2.50 (residual DMSO), 2.20 (2H, t, *J* = 7.2Hz, C2-H).

^{13}C NMR (100 MHz, DMSO-d_6): δ 168.8(C1), 158.0(Ar$\underline{C}$-OCH_3),133.3(ArC), 129.8(ArCH), 114.1(ArCH), 55.4(O$\underline{C}H_3$), 34.7(C3), 30.4(C2).

HRMS (TOF MS ES+): *m/z* $[M + Na]^+$ calcd. for $C_{10}H_{13}NNaO_3$ 218.0793; found 218.0788.

6-(Hydroxyamino)-6-oxohexanoic acid (7h):

Eluent: $CHCl_3$:MeOH (95:5).

Yield: 81%. Colorless viscous liquid.

IR (neat): 3202, 2933, 1710, 1656, 1461cm^{-1}

^{1}H NMR (400 MHz, DMSO-d_6): δ 10.40 (1H, brs, NH), 3.41 (H_2O from DMSO-d6),3.17 (solvent impurity), 2.50 (residual DMSO), 2.17 (2H, t, J = 6.4 Hz, C2-H), 1.94 (2H, t, J = 6.4 Hz, C5-H), 1.47 (4H, brs, C3-H+C4-H).

^{13}C NMR (100 MHz, DMSO-d_6): δ175.3 (C1), 169.8(C6), 34.1(C2), 32.4(C5), 25.1(C4), 24.8(C3).

HRMS (TOF MS ES+): *m/z* $[M + Na]^+$ calcd. for $C_6H_{11}NNaO_4$ 184.0586; found 184.0598

7-(Hydroxyamino)-7-oxoheptanoic acid (7i):

Eluent: $CHCl_3$:MeOH (95:5).

Yield: 78%. Colorless viscous liquid.

IR (neat): 3419, 2925, 2855, 2256, 2127, 1651cm^{-1}

^{1}H NMR (400 MHz, DMSO-d_6): δ 10.42 (1H, s, NH), 3.54 (H_2O from DMSO-d6), 2.59 (residual DMSO), 2.27 (2H, t, *J* = 7.2Hz, C2-H), 2.02 (2H, t, *J* = 7.2Hz, C6-H), 1.60-1.53 (4H, m. C3-H+C5-H), 1.35-1.32 (2H, m, C4-H).

^{13}C NMR (100 MHz, DMSO-d_6): δ 174.9(C1), 169.6(C7), 34.0(C2), 32.6(C6), 28.4(C4), 25.3(C5), 24.7(C3).

HRMS (TOF MS ES+): *m/z* [M + Na]$^+$ calcd. for $C_7H_{13}NNaO_4$ 198.0742; found 198.0748.

8-(Hydroxyamino)-8-oxooctanoic acid (7j):

Eluent: $CHCl_3$:MeOH (95:5).

Yield: 75%. Colorless viscous liquid.

IR (neat): 3414, 2922, 2855, 1637 cm^{-1}.

^{1}H NMR (400 MHz, DMSO-d_6): δ 12.06 (1H, brs, COO<u>H</u>), 10.40 (1H, s. NH), 8.74 (1H, brs, OH), 3.43 (H_2O from DMSO-d6), 2.57 (residual DMSO), 2.25 (2H, t, *J* = 6.8Hz, C2-H), 1.99 (2H, t, *J* = 7.2Hz, C7-H), 1.53 (4H, brs, C3-H+C-6H), 1.30 (4H, brs, C4-H+C5-H).

^{13}C NMR (100 MHz, DMSO-d_6): δ 175.0(C1), 169.6(C8), 34.1(C2), 32.7(C7), 28.7(two signals, C4+C5), 25.4(C6), 24.9(C3).

HRMS (TOF MS ES+): *m/z* [M + Na]$^+$ calcd. for $C_8H_{15}NNaO_4$ 212.0899; found 212.0892.

(*S*)-Methyl 2-((*tert*-butoxycarbonyl)amino)-7-(hydroxyamino)-7-oxoheptanoate (7k) :

Eluent: $CHCl_3$:MeOH (97:3).

Yield: 86%. Colorless viscous liquid.

$[\alpha]_D^{25}$= +4.2 (c 2.5, $CHCl_3$).

IR (neat): 3414, 3320, 2927, 2855, 1744, 1694 cm^{-1}.

^{1}H NMR (400 MHz, DMSO-d_6): δ 10.35 (1H, s, NH), 8.69 (1H, brs, OH), 7.25 (1H, d, *J* = 8Hz, N<u>H</u>Boc), 3.92 (1H, m, C2-H), 3.62 (3H, s, $COOCH_3$), 3.61 (H_2O from DMSO-d6), 2.50 (residual DMSO), 1.95 (2H, m, C6-H), 1.62-1.52 (6H, m, C3-H+C4-H+C5-H), 1.38 (9H, s, O-CMe_3).

^{13}C NMR (100 MHz, $CDCl_3$): δ 173.5 (C1), 169.1(C7) 156.0 (<u>C</u>OOCMe$_3$), 78.7(O<u>C</u>Me$_3$), 53.7 (CO_2<u>Me</u>), 52.4(C2), 32.1(C6), 30.6(C3), 28.6(OC<u>Me$_3$</u> + C-3, two signals), 22.2(C4).

HRMS (TOF MS ES+): *m/z* $[M + Na]^+$ calcd. for $C_{13}H_{24}N_2NaO_6$ 327.1532; found 327.1539.

(*S*)-Methyl 2-((*tert*-butoxycarbonyl)amino)-8-(hydroxyamino)-8-oxooctanoate (7l):

Eluent: $CHCl_3$:MeOH (97:3).

Yield: 84%. Colorless viscous liquid.

$[\alpha]_D^{25}$= + 3.84 (c 1.25, $CHCl_3$).

IR (neat): 3429, 3289, 2947, 1744, 1694, 1533 cm^{-1}.

1H NMR (400 MHz, $CDCl_3$): δ 5.19 (1H, brs, N<u>H</u>Boc), 4.18 (1H, s, C2-H), 3.67 (3H, s, CO_2<u>Me</u>), 2.09 (2H, m, C7-H), 1.68-1.49 (4H, m, C3-H+C6-H), 1.36 (9H, s, CO_2C<u>Me</u>$_3$), 1.26-1.21 (4H, m, C4-H+C5-H).

^{13}C NMR (100 MHz, $CDCl_3$): δ 173.5(C1), 155.8($\underline{C}OOCMe_3$), 80.2 ($O\underline{C}Me_3$), 53.2($CO_2\underline{Me}$), 52.3(C2), 32.4(C7), 29.7(C3), 28.3($OC\underline{Me_3}$), 28.1(C5), 24.9(C4), 24.7(C6).

HRMS (TOF MS ES+): *m/z* $[M + Na]^+$ calcd. for $C_{14}H_{26}N_2NaO_6$ 341.1688; found 341.1689

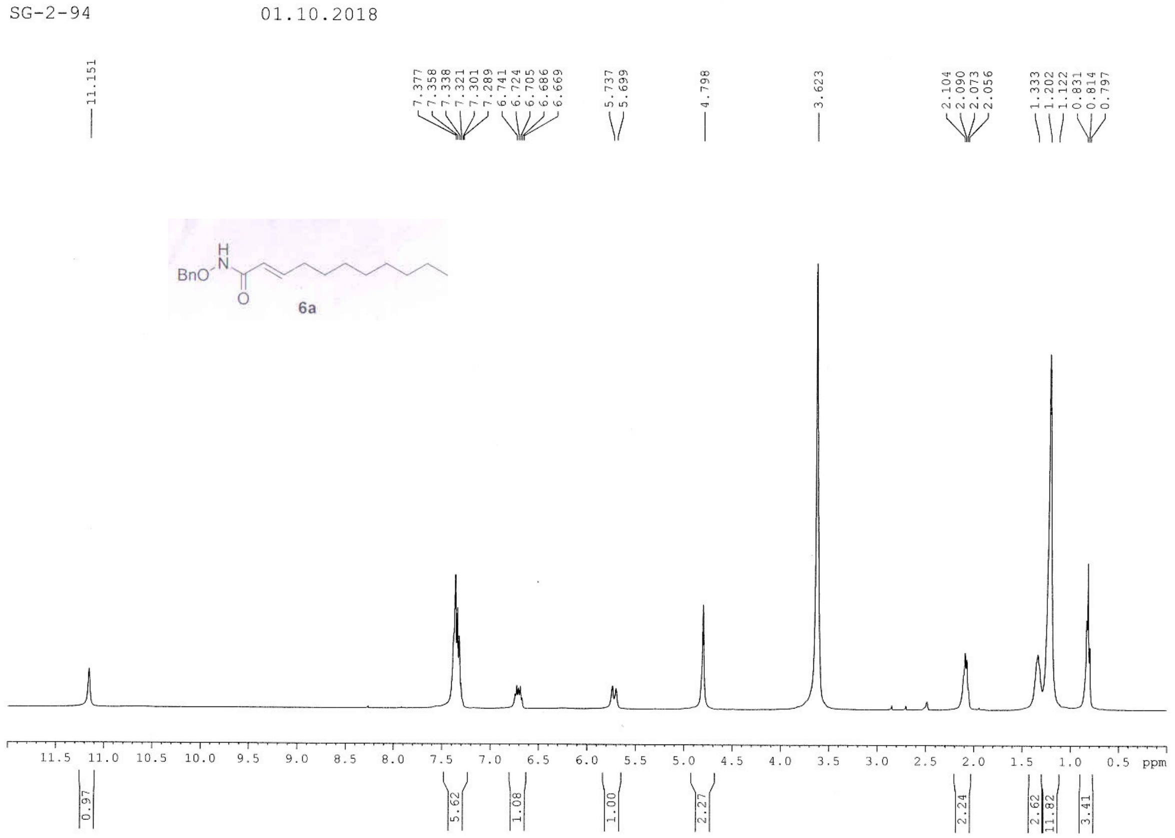

^{1}H NMR Spectrum of compound **6a** in DMSO-d_6

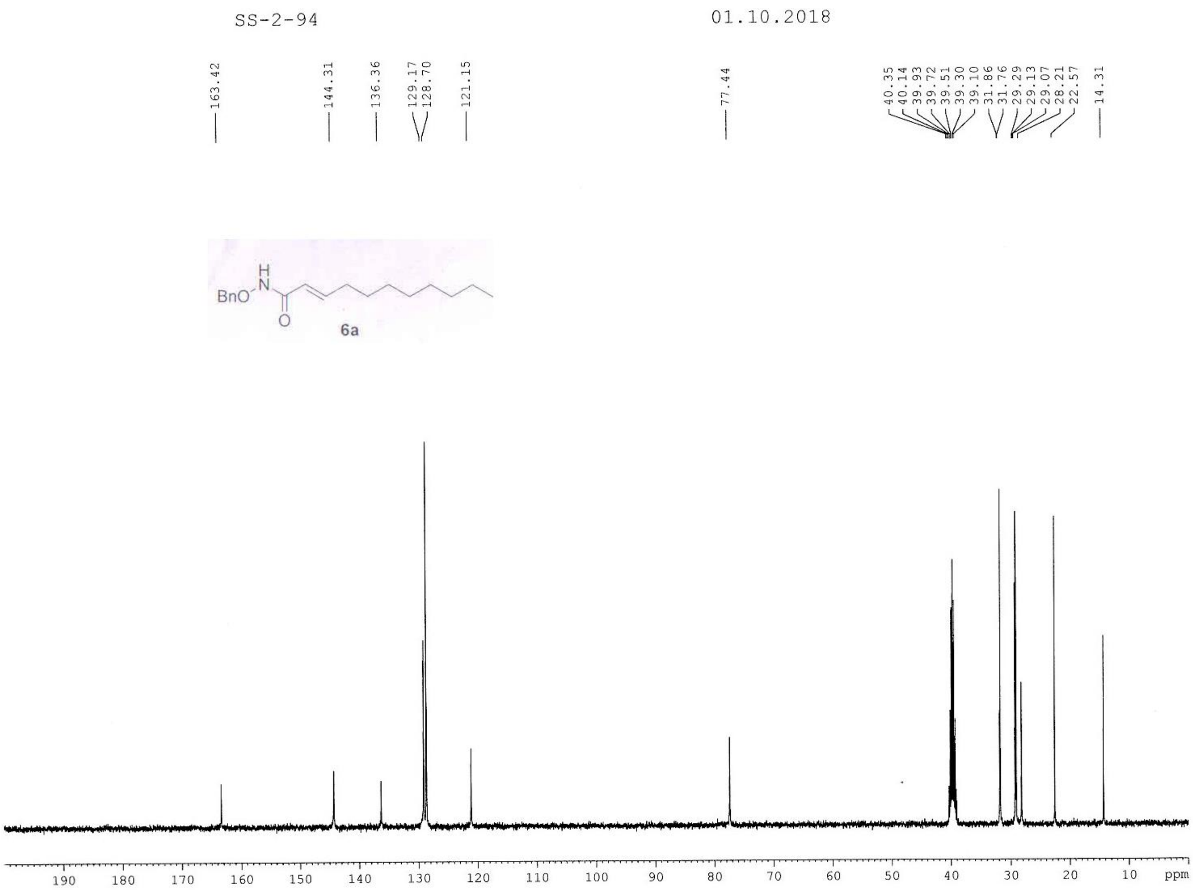

^{13}C NMR Spectrum of compound **6a** in DMSO- d_6

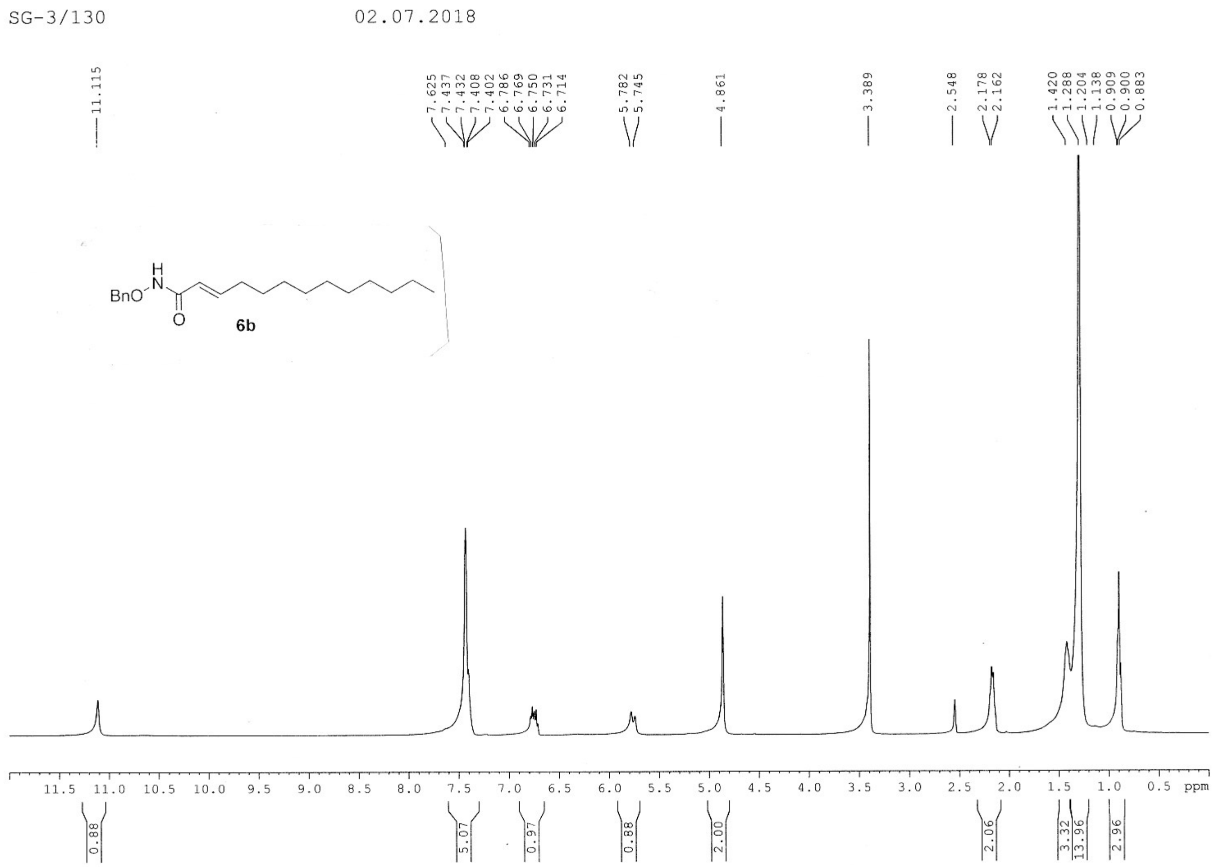

^{1}H NMR Spectrum of compound **6b** in DMSO-d_6

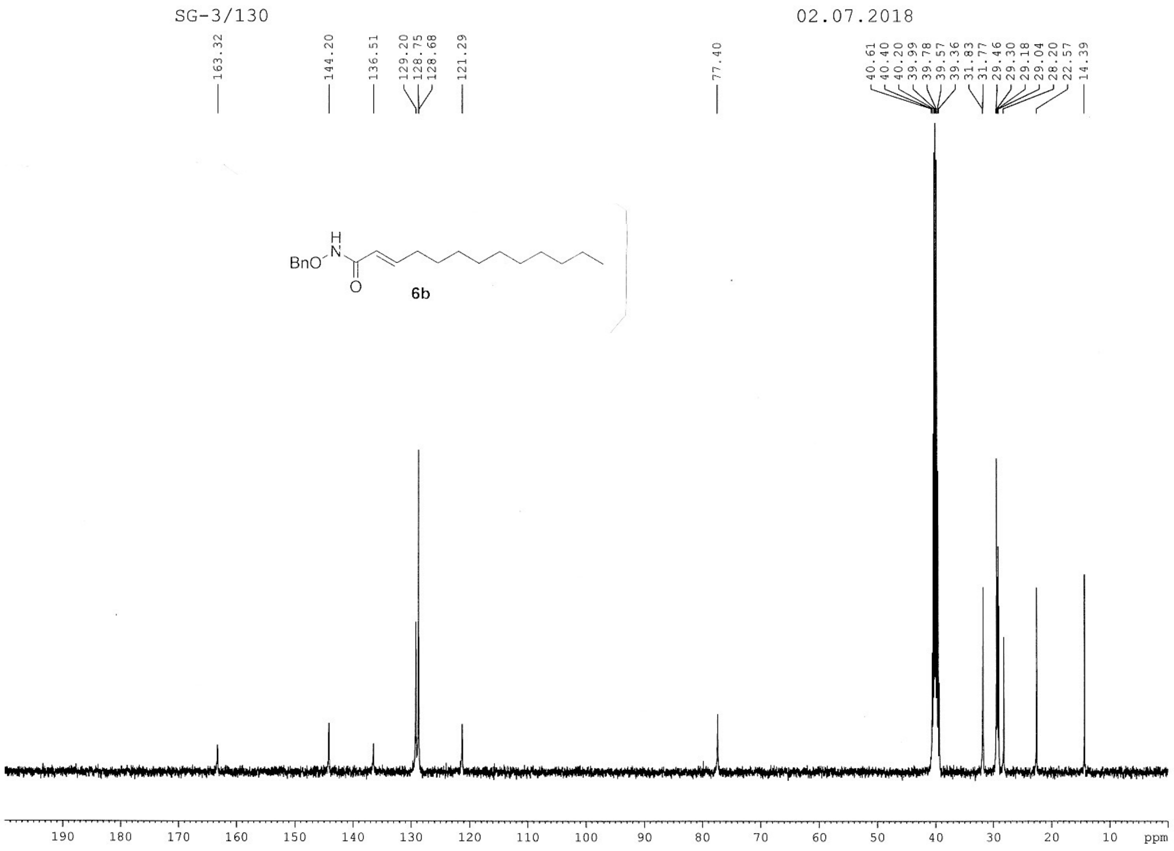

^{13}C NMR Spectrum of compound **6b** in DMSO-d_6

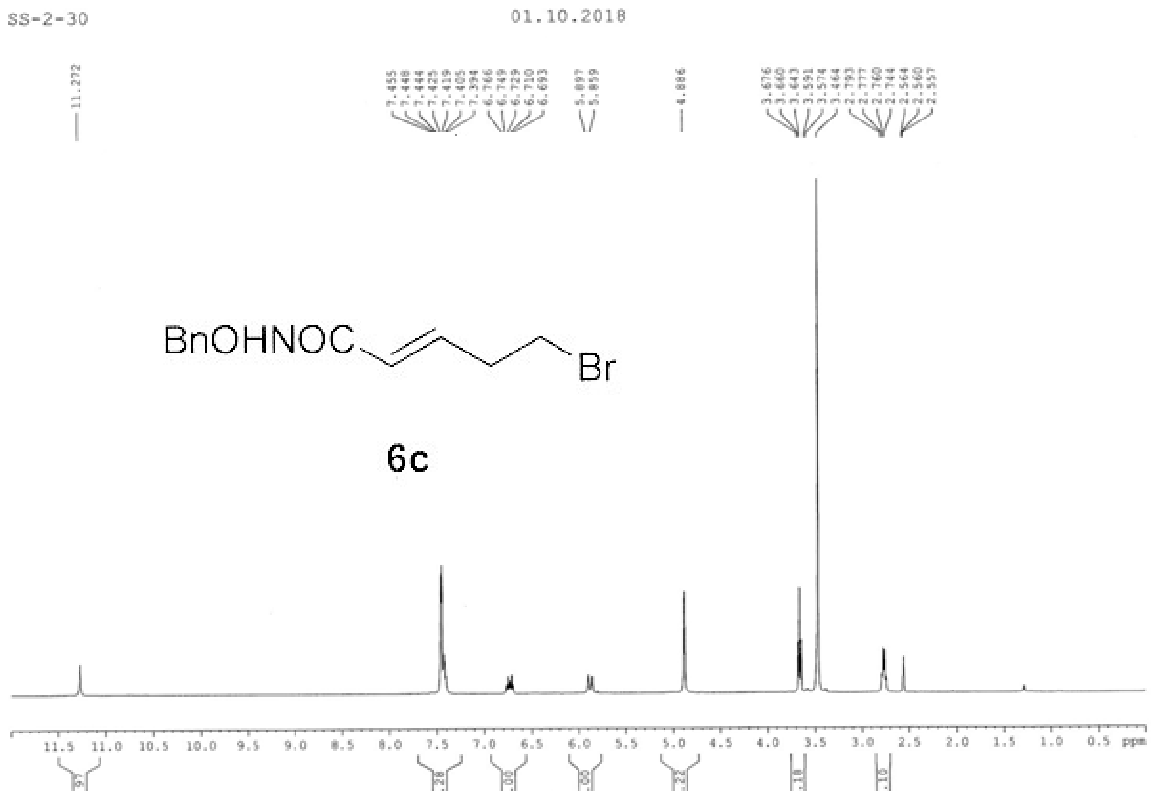

^{1}H NMR Spectrum of compound **6c** in DMSO-d_6

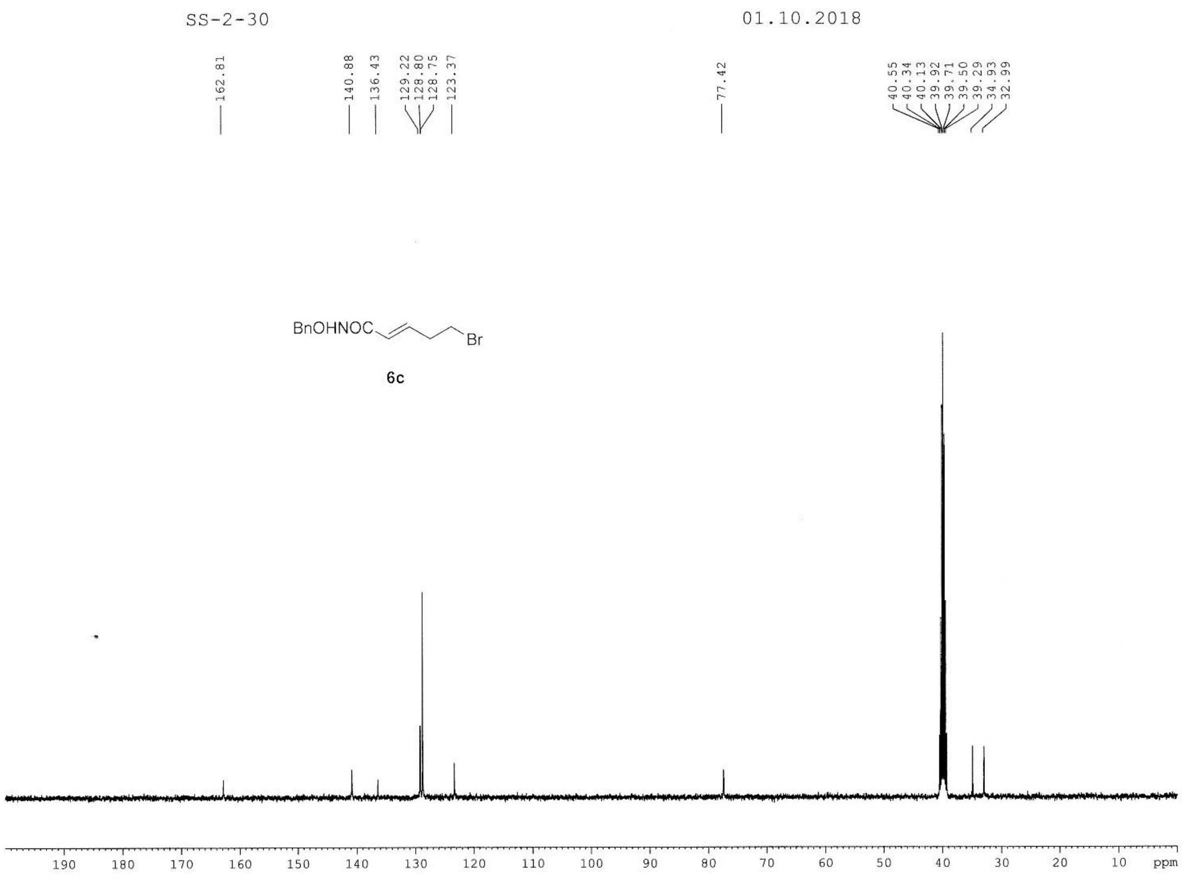

^{13}C NMR Spectrum of compound **6c** in DMSO-d_6

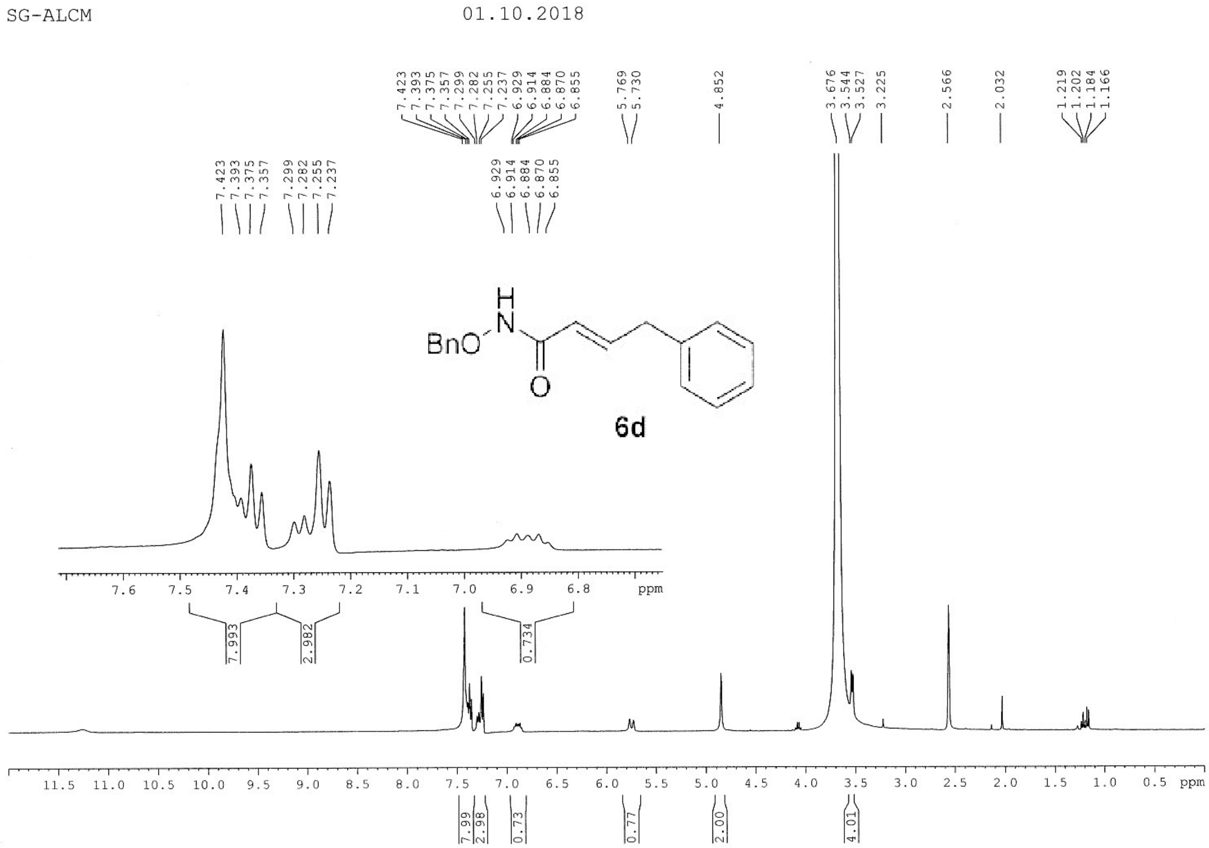

^{1}H NMR Spectrum of compound **6d** in DMSO-d_6

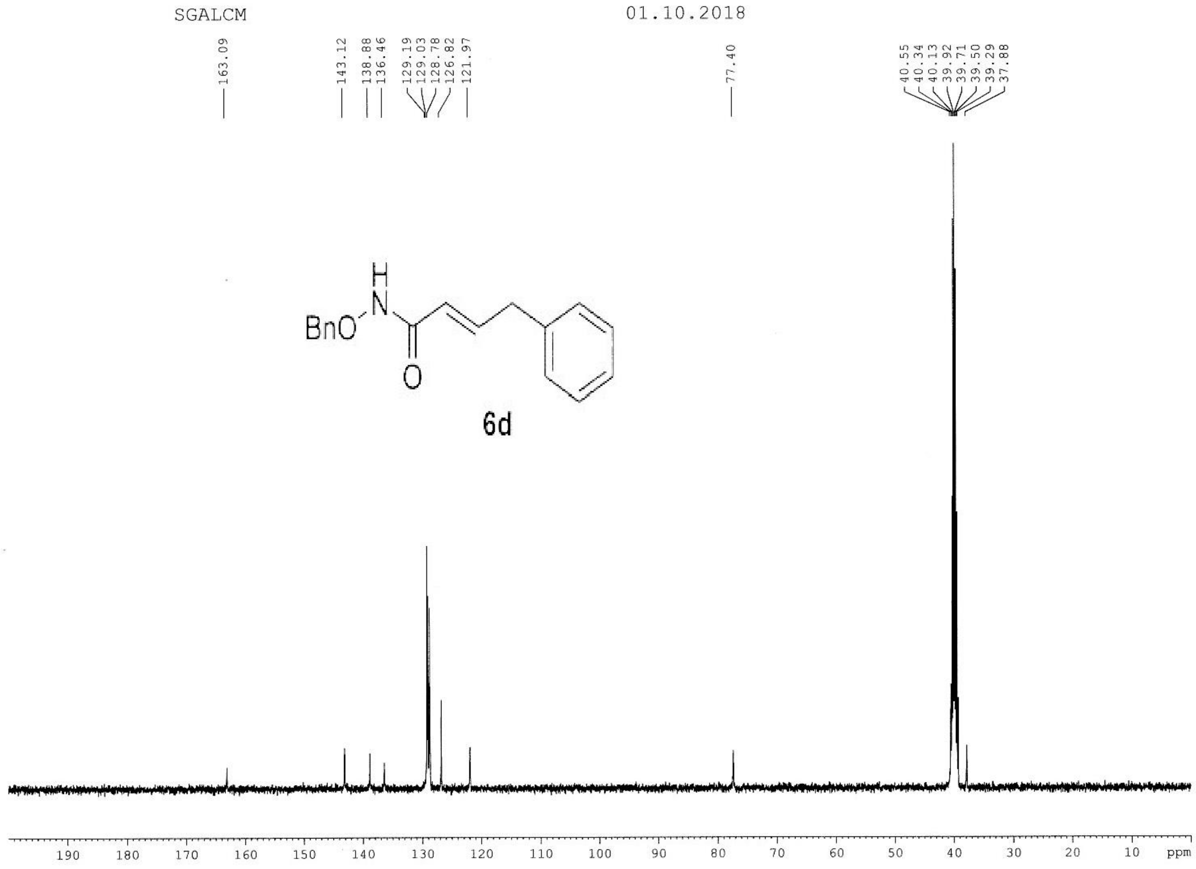

^{13}C NMR Spectrum of compound **6d** in DMSO-d_6

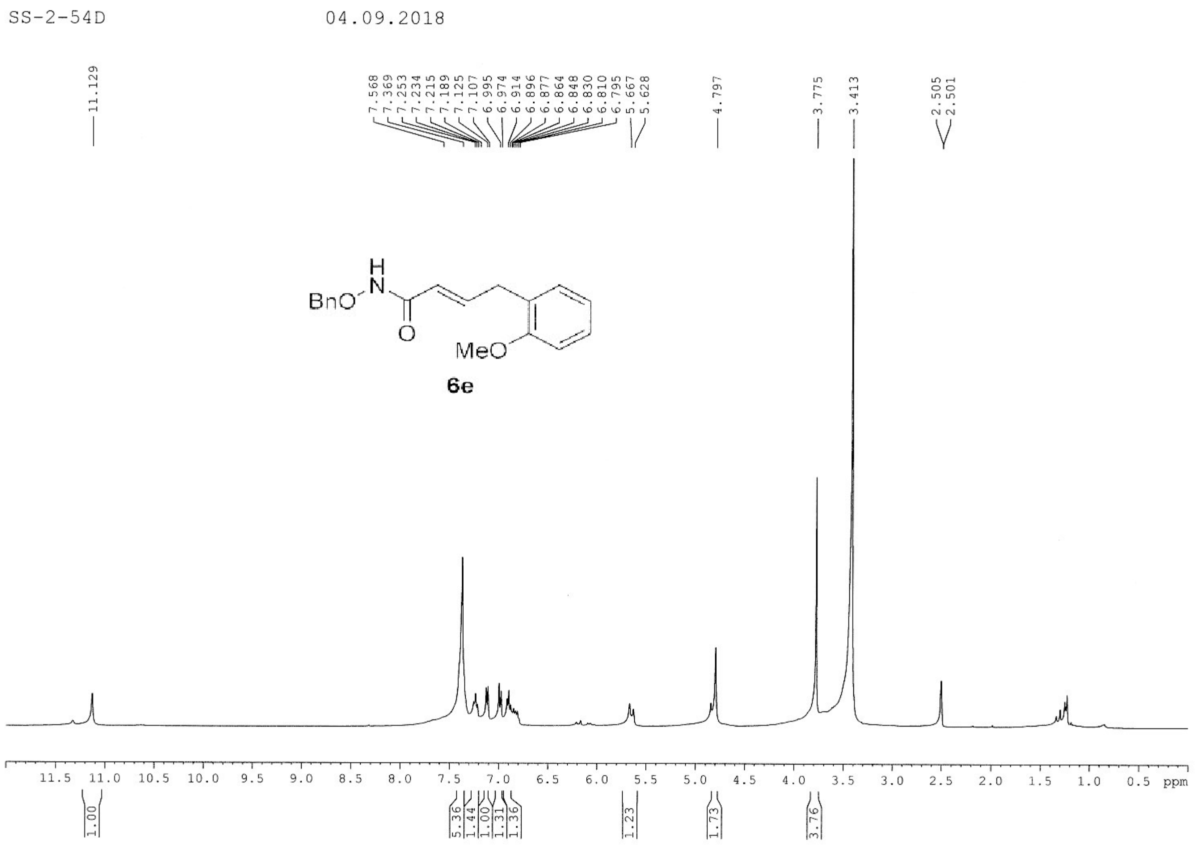

^{1}H NMR Spectrum of compound **6e** in DMSO-d_6

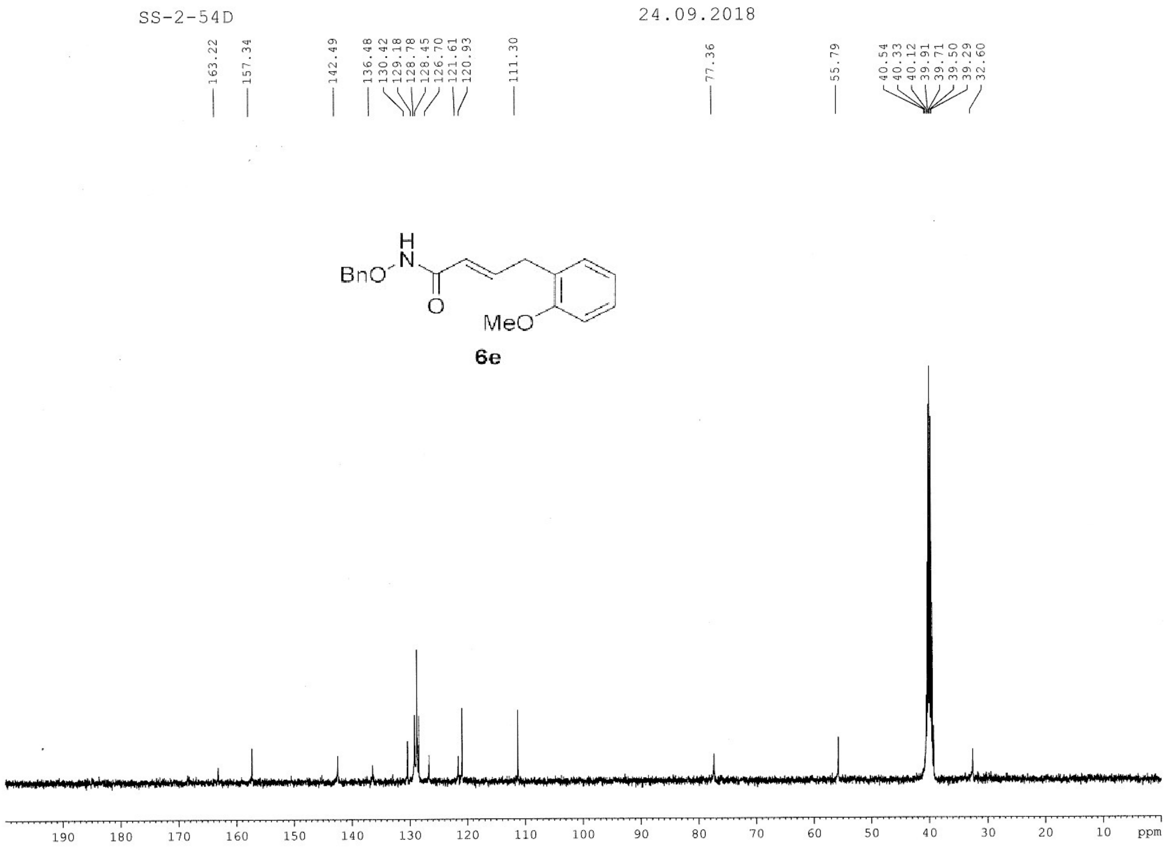

^{13}C NMR Spectrum of compound **6e** in DMSO-d_6

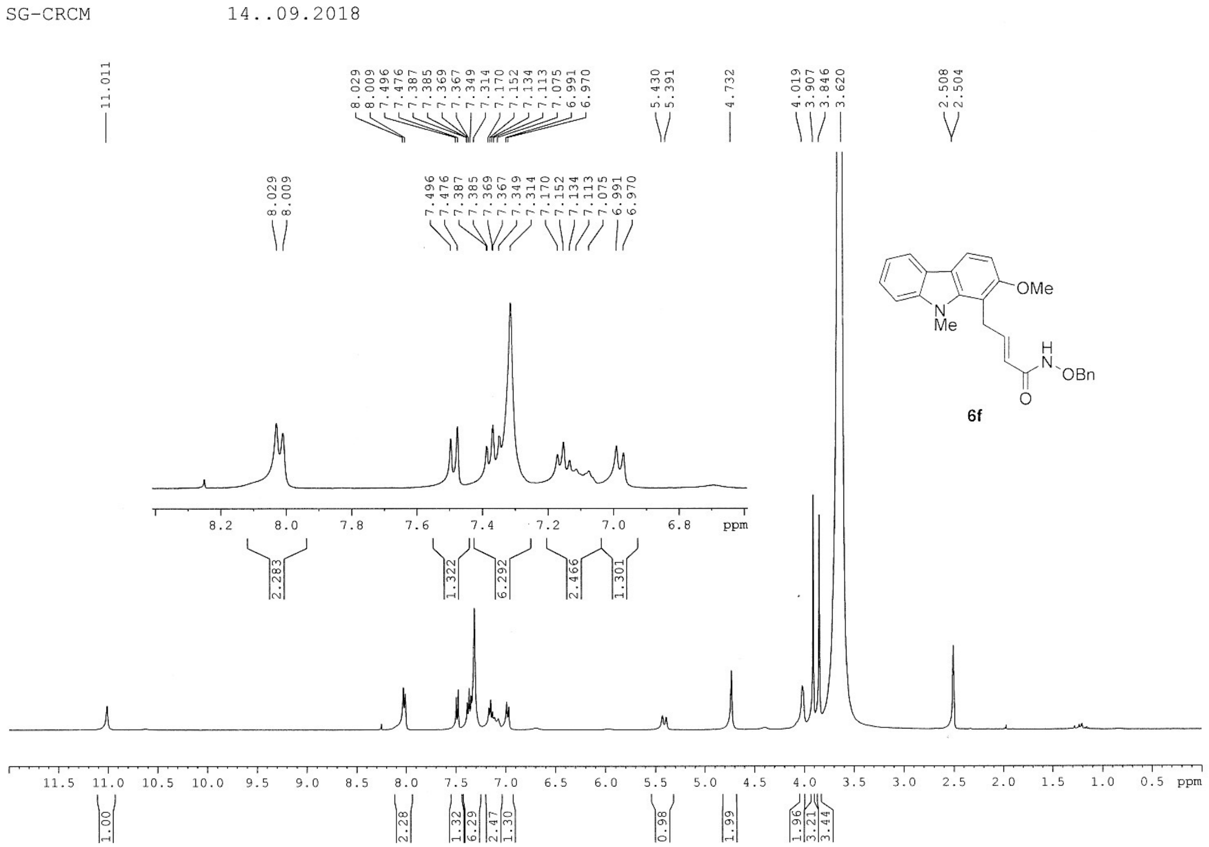

^{1}H NMR Spectrum of compound **6f** in DMSO-d_6

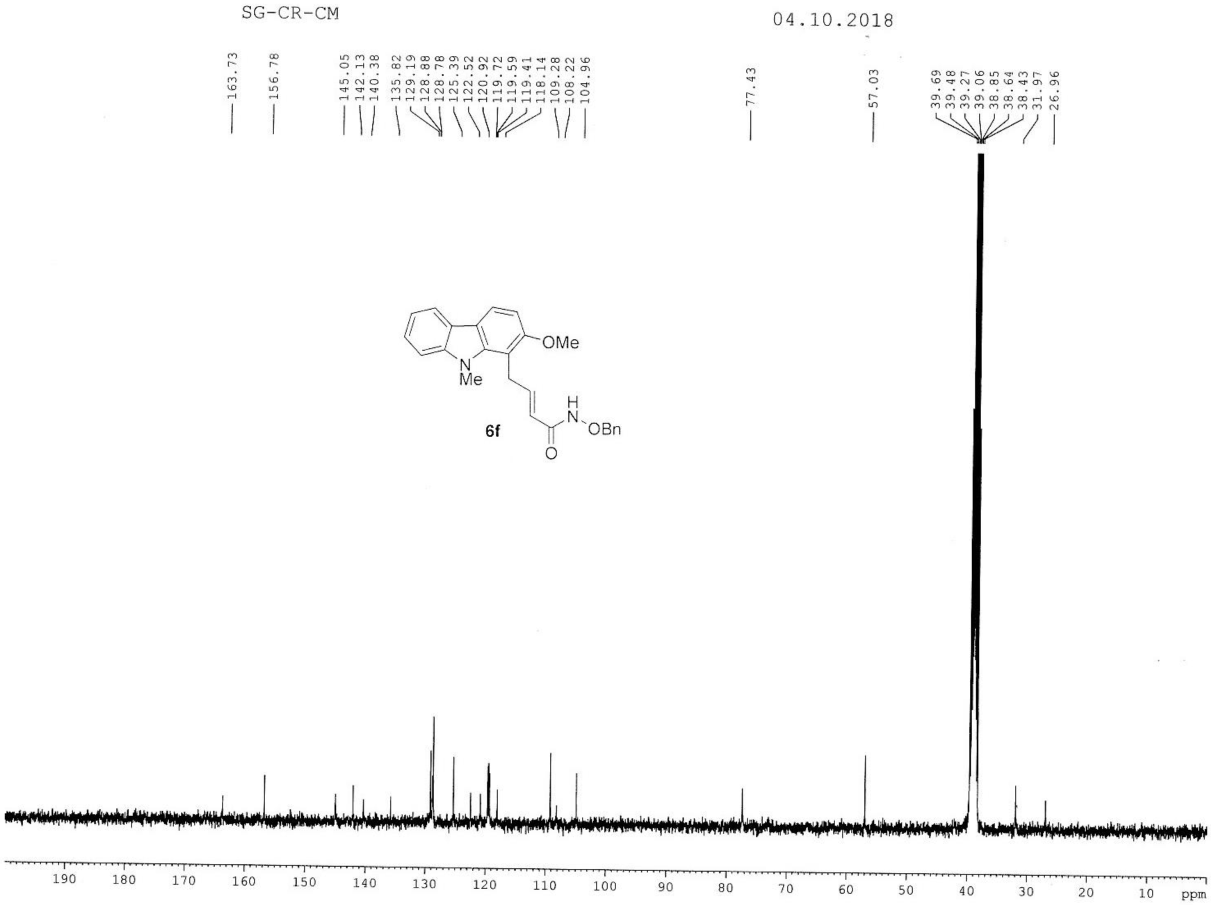

^{13}C NMR Spectrum of compound **6f** in DMSO-d_6

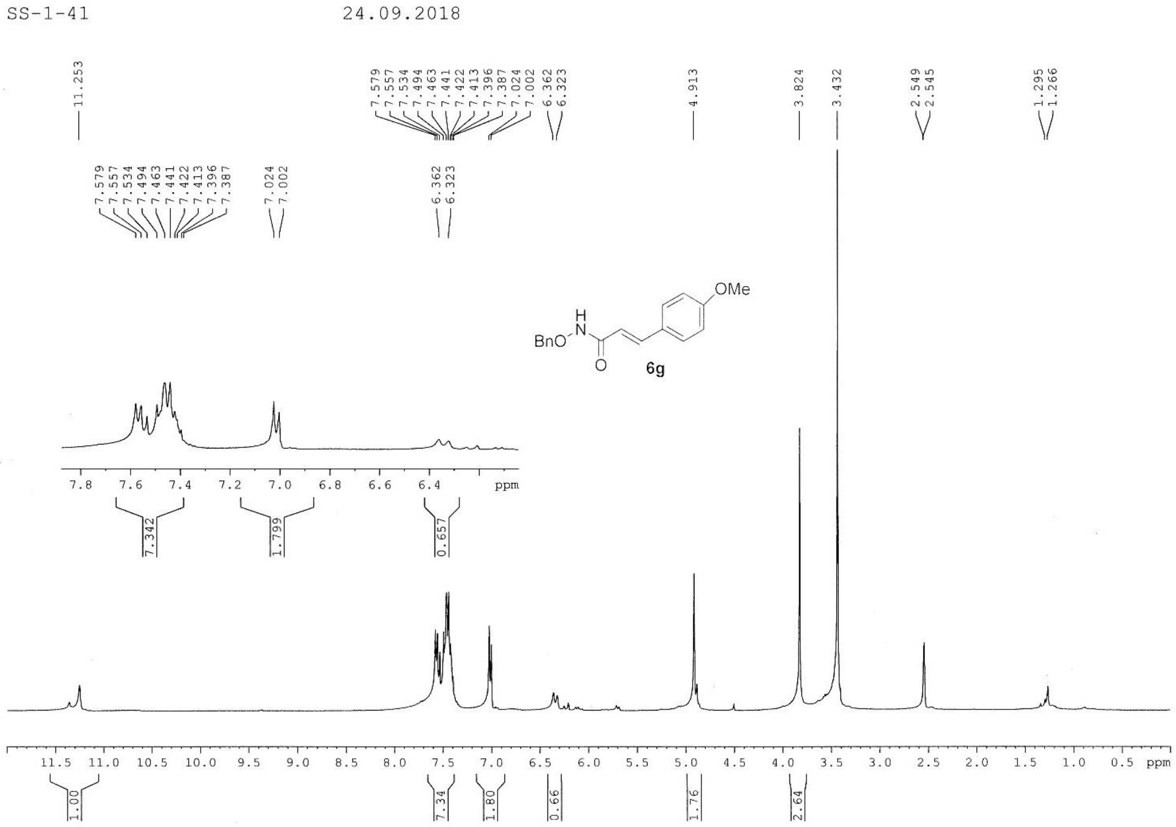

^{1}H NMR Spectrum of compound **6g** in DMSO-d_6

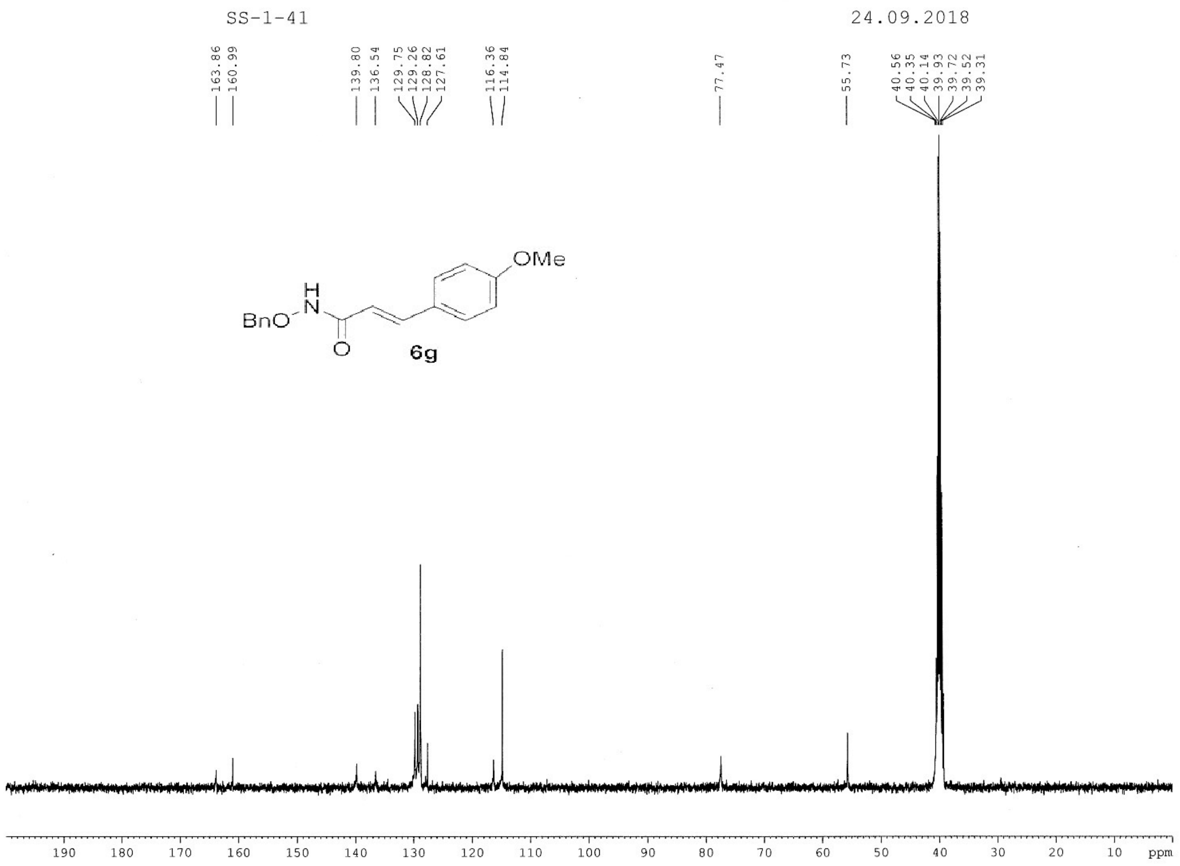

^{13}C NMR Spectrum of compound **6g** in DMSO-d_6

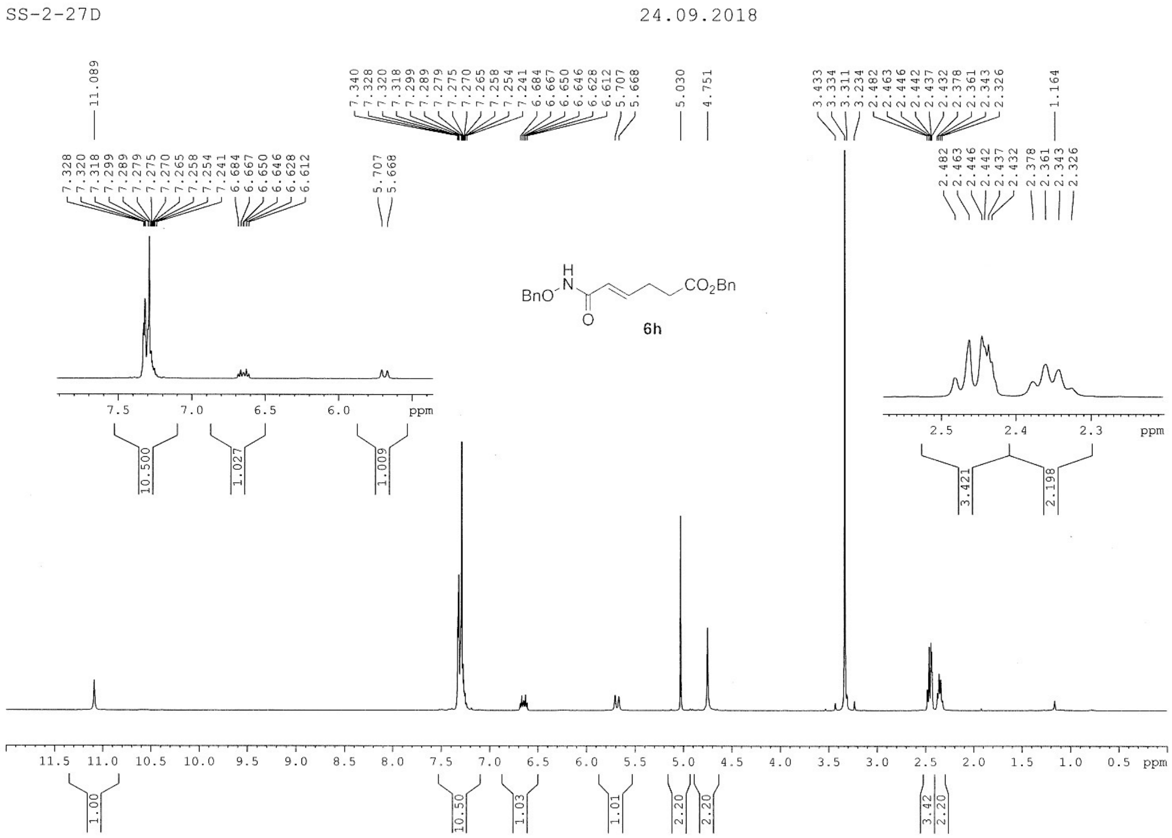

^{1}H NMR Spectrum of compound **6h** in DMSO-d_6

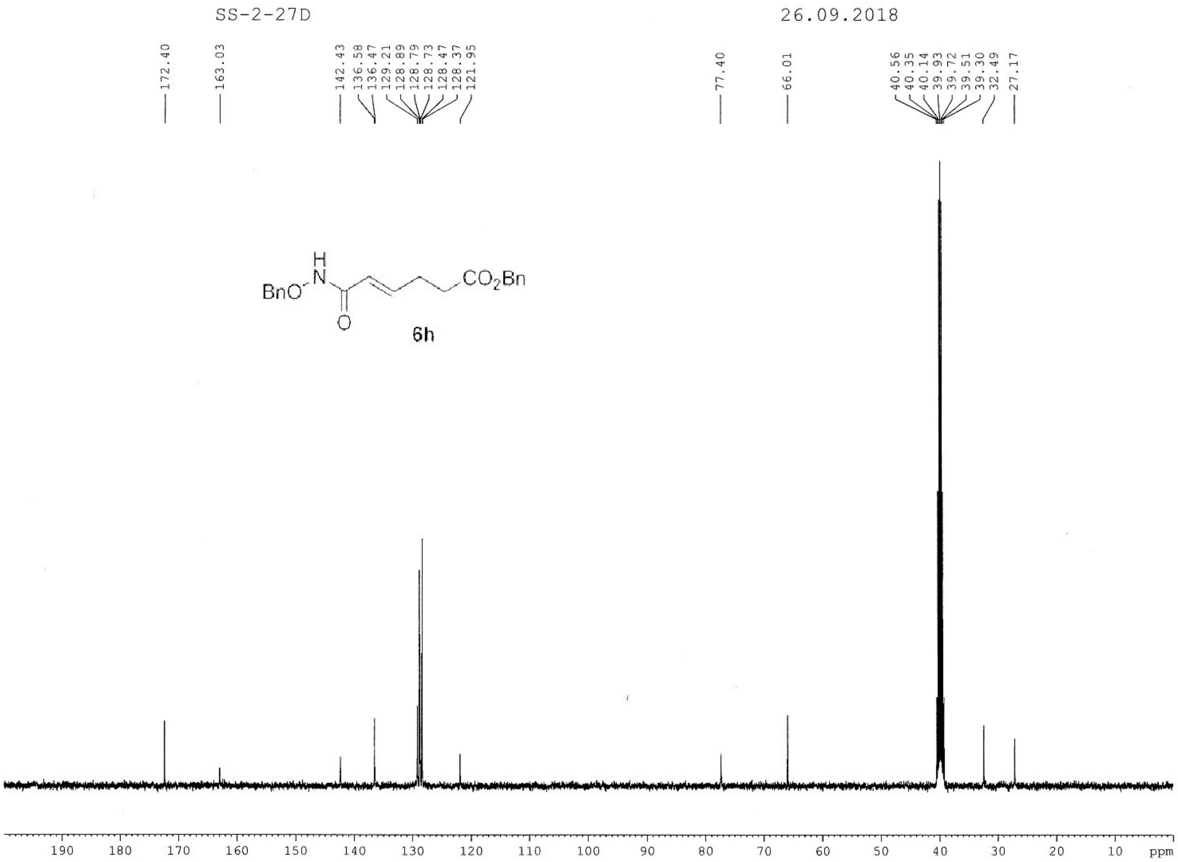

^{13}C NMR Spectrum of compound **6h** in DMSO-d_6

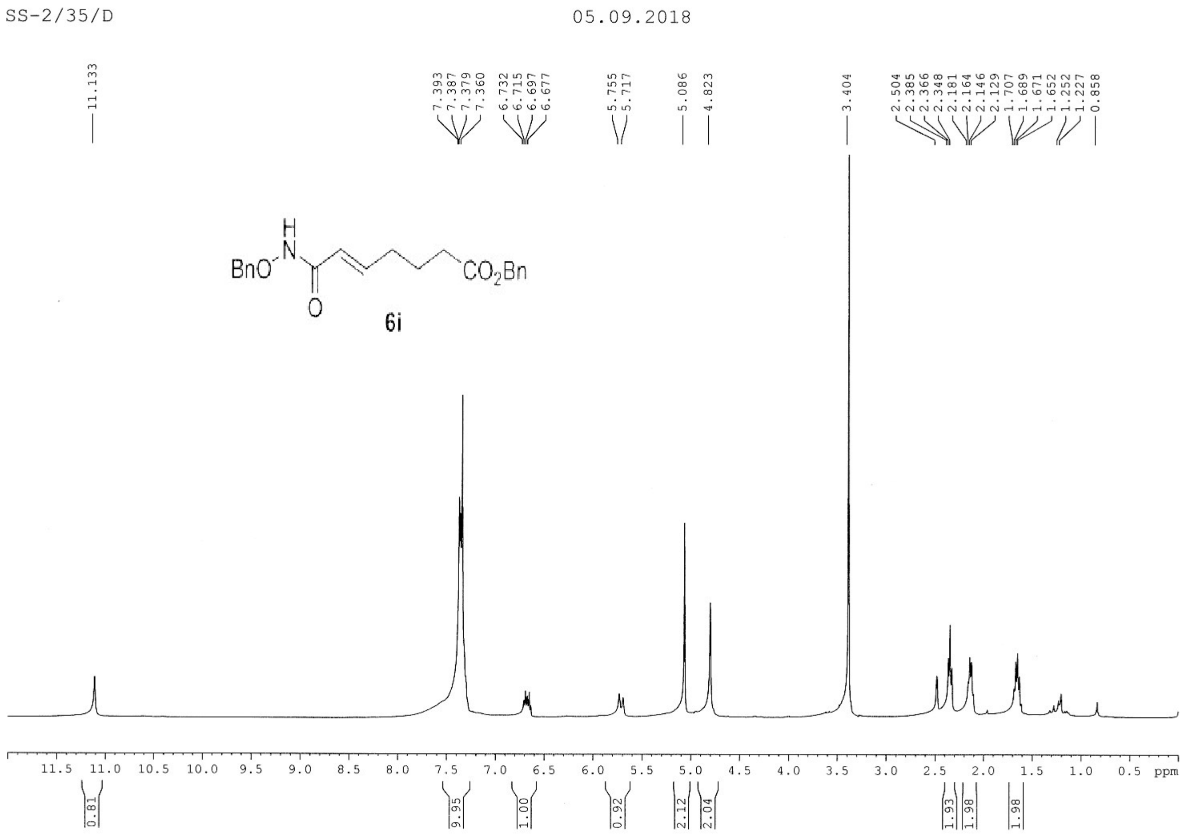

^{1}H NMR Spectrum of compound **6i** in DMSO-d_6

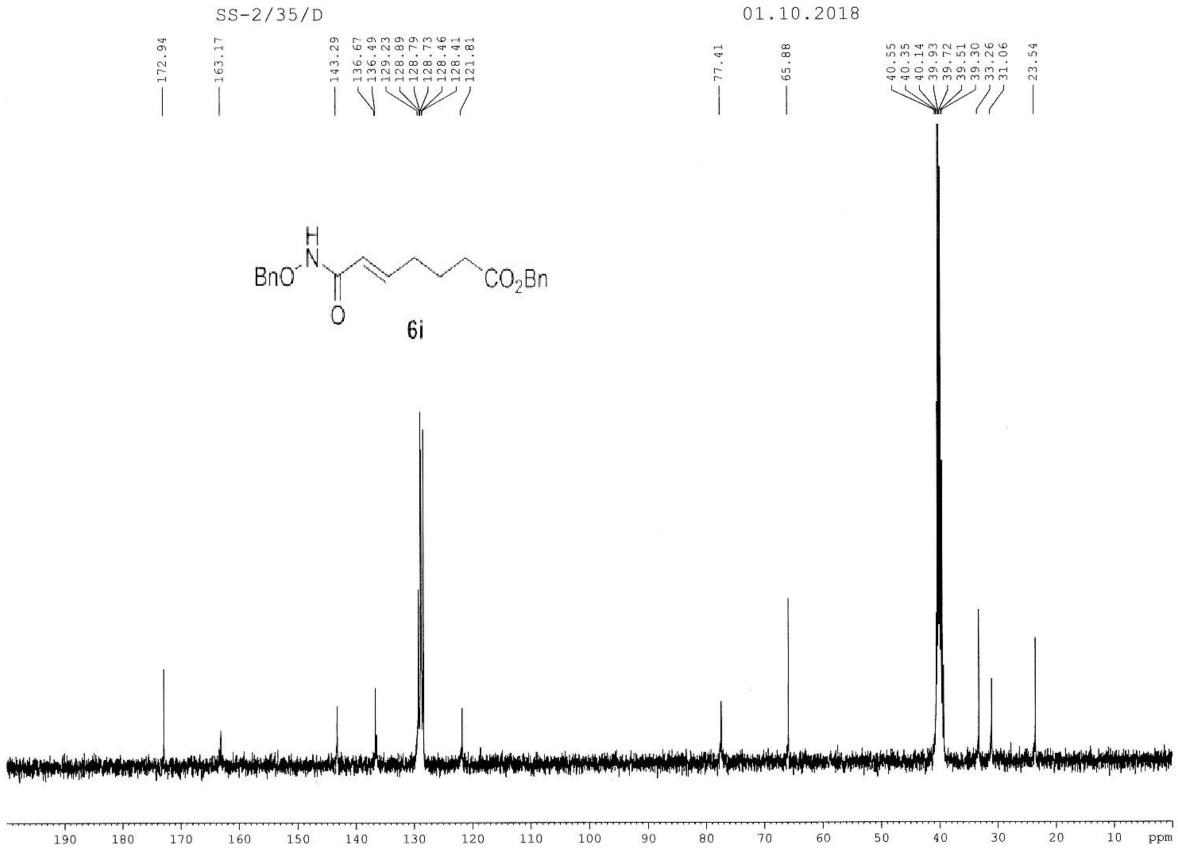

^{13}C NMR Spectrum of compound **6i** in DMSO-d_6

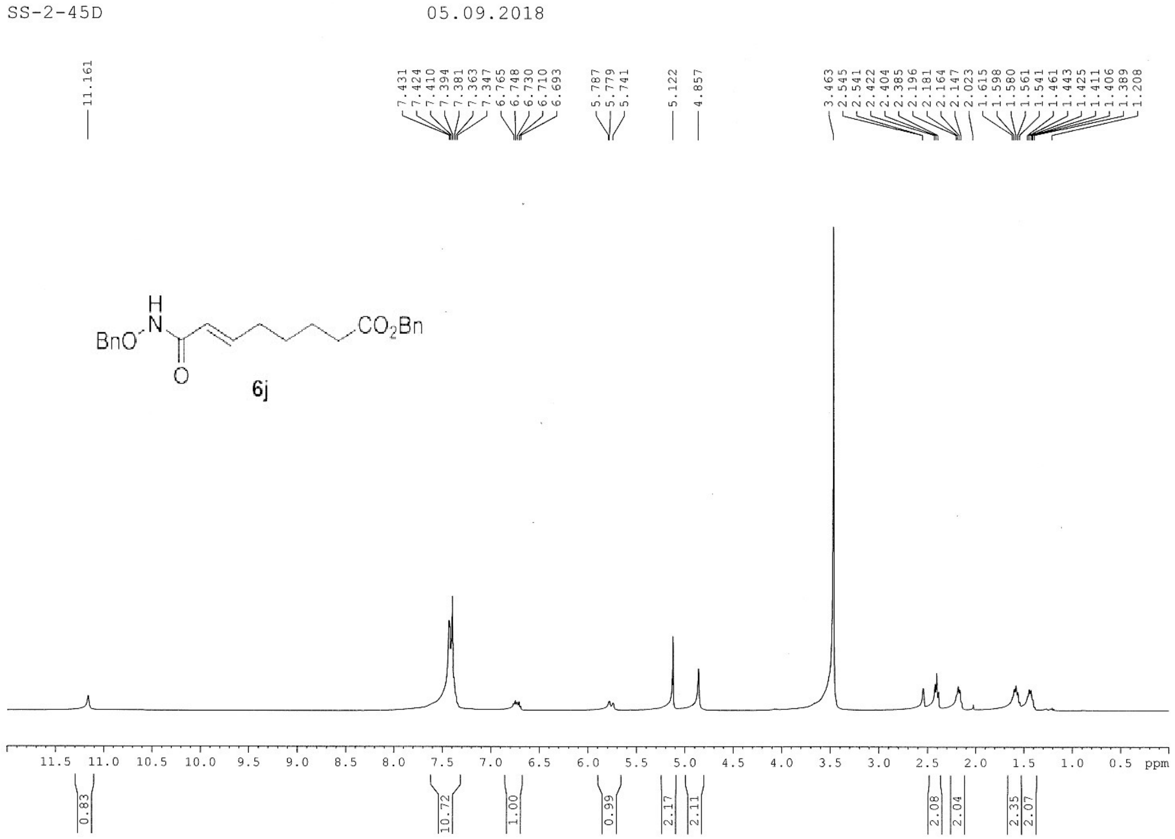

^{1}H NMR Spectrum of compound **6j** in DMSO-d_6

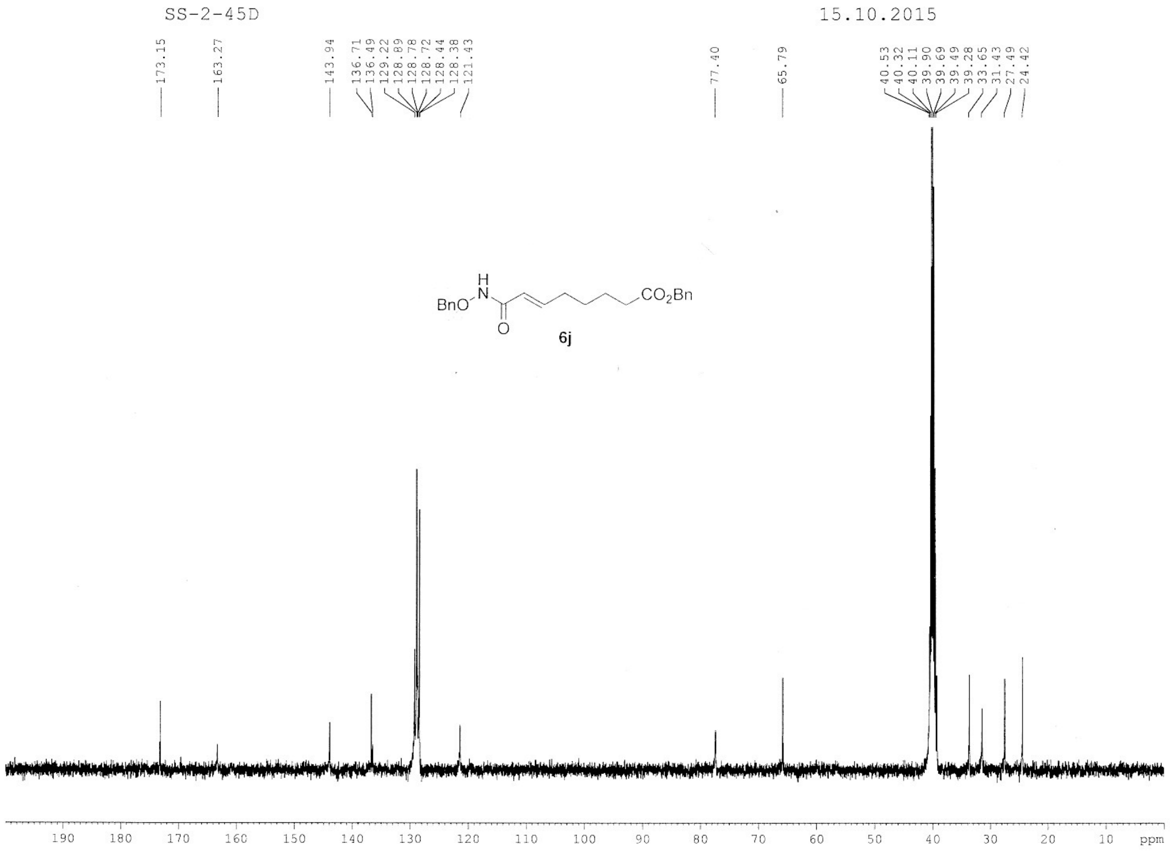

^{13}C NMR Spectrum of compound **6j** in DMSO-d_6

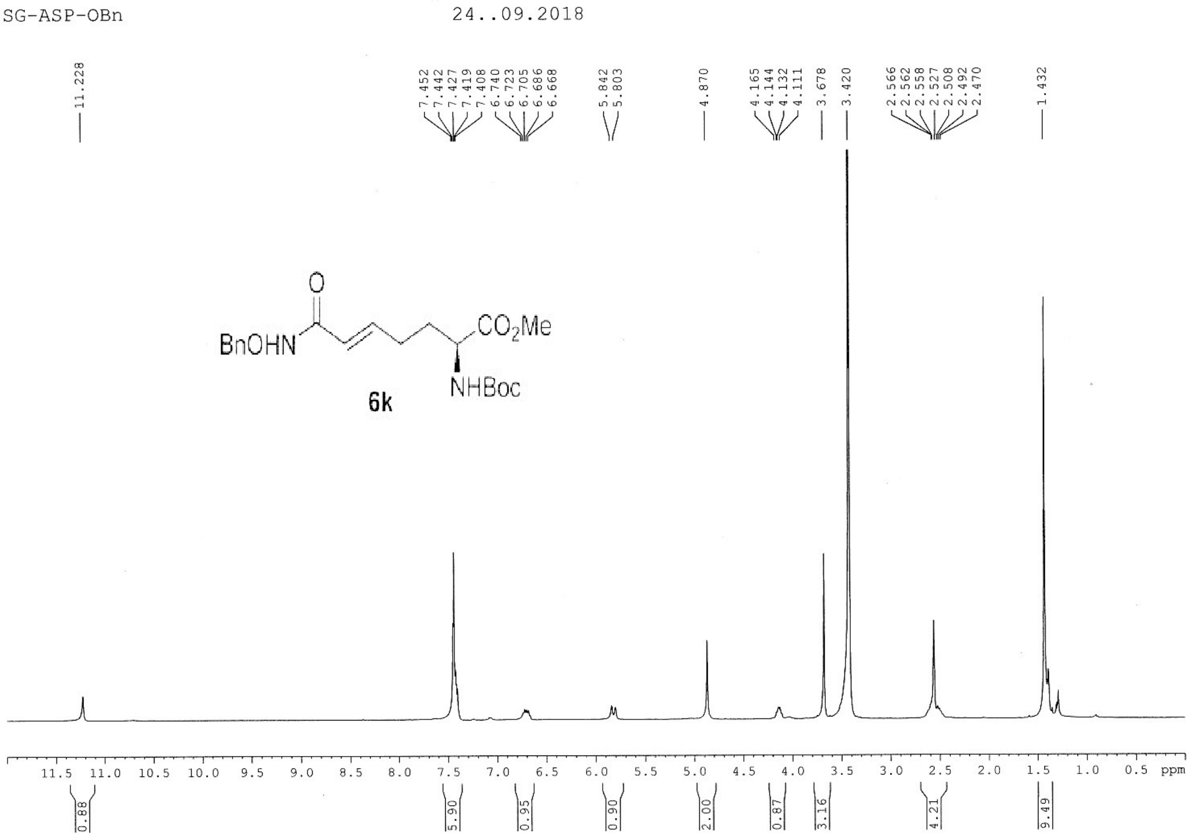

^{1}H NMR Spectrum of compound **6k** in DMSO-d_6

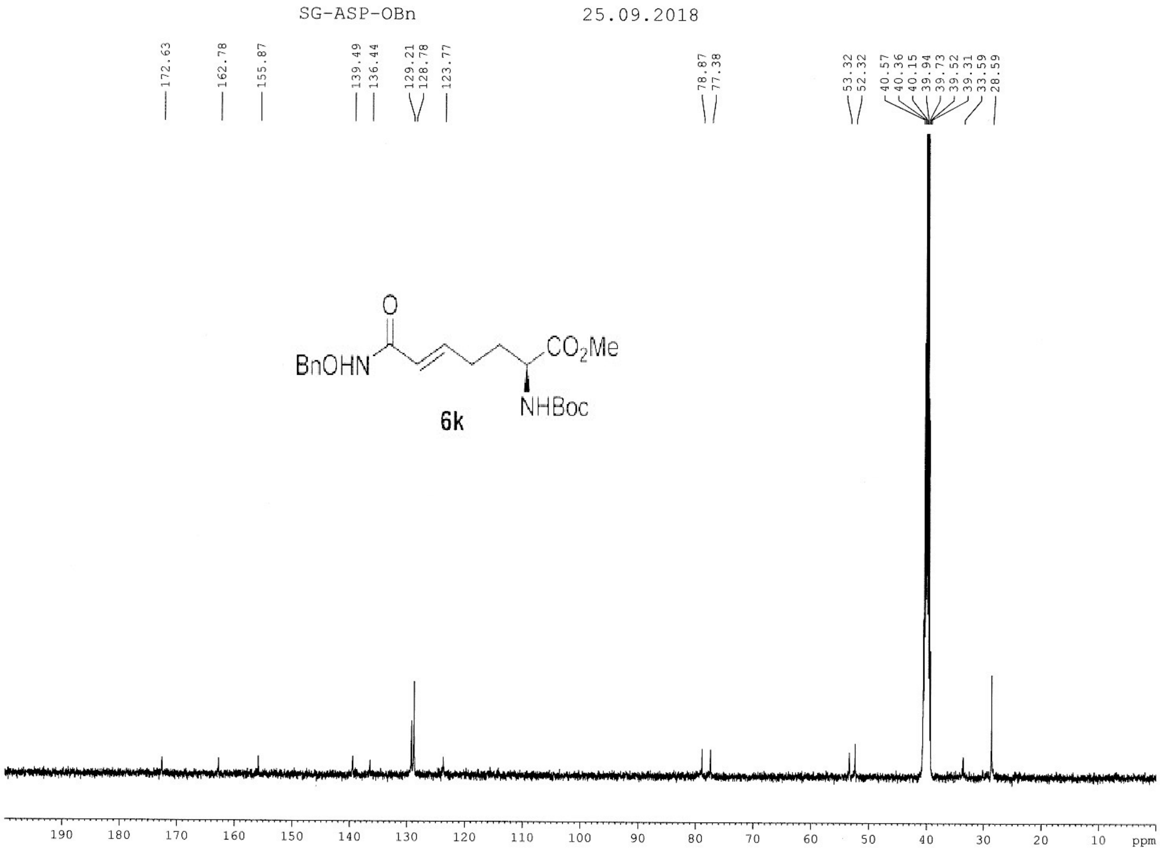

^{13}C NMR Spectrum of compound **6k** in DMSO-d_6

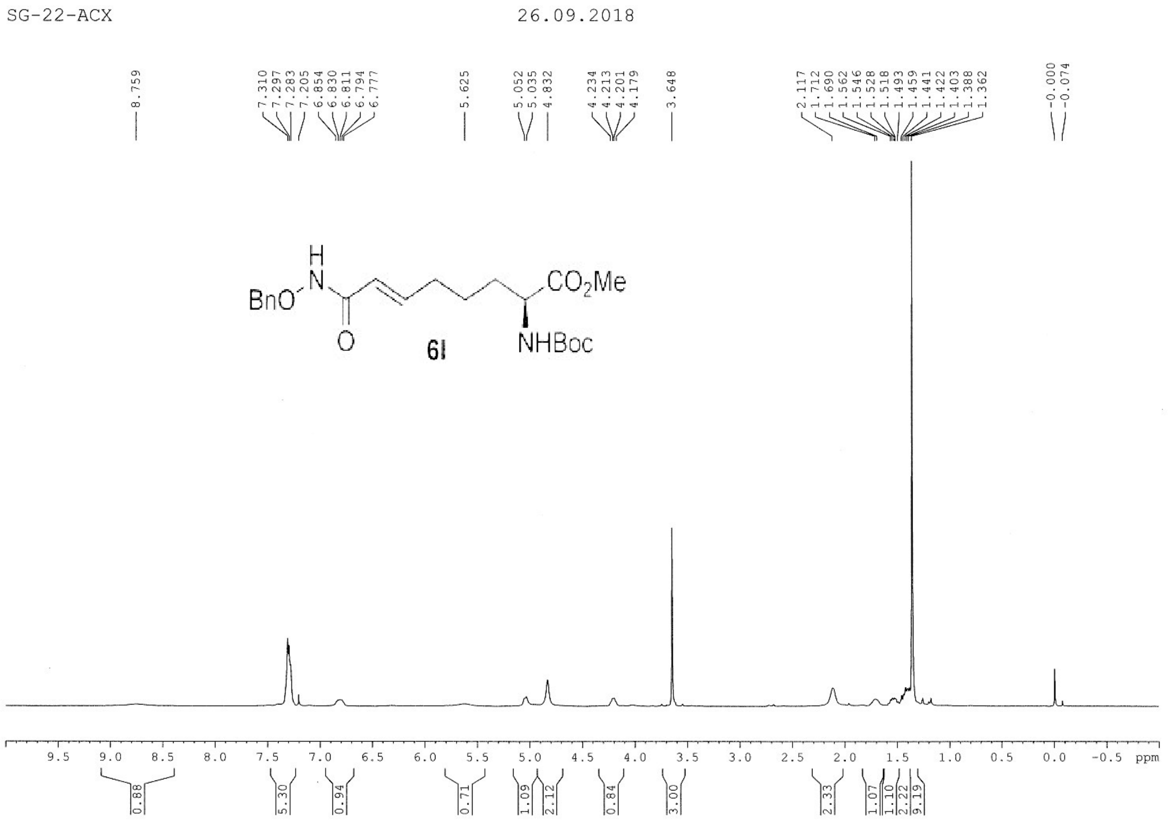

^{1}H NMR Spectrum of compound **6l** in DMSO-d_6

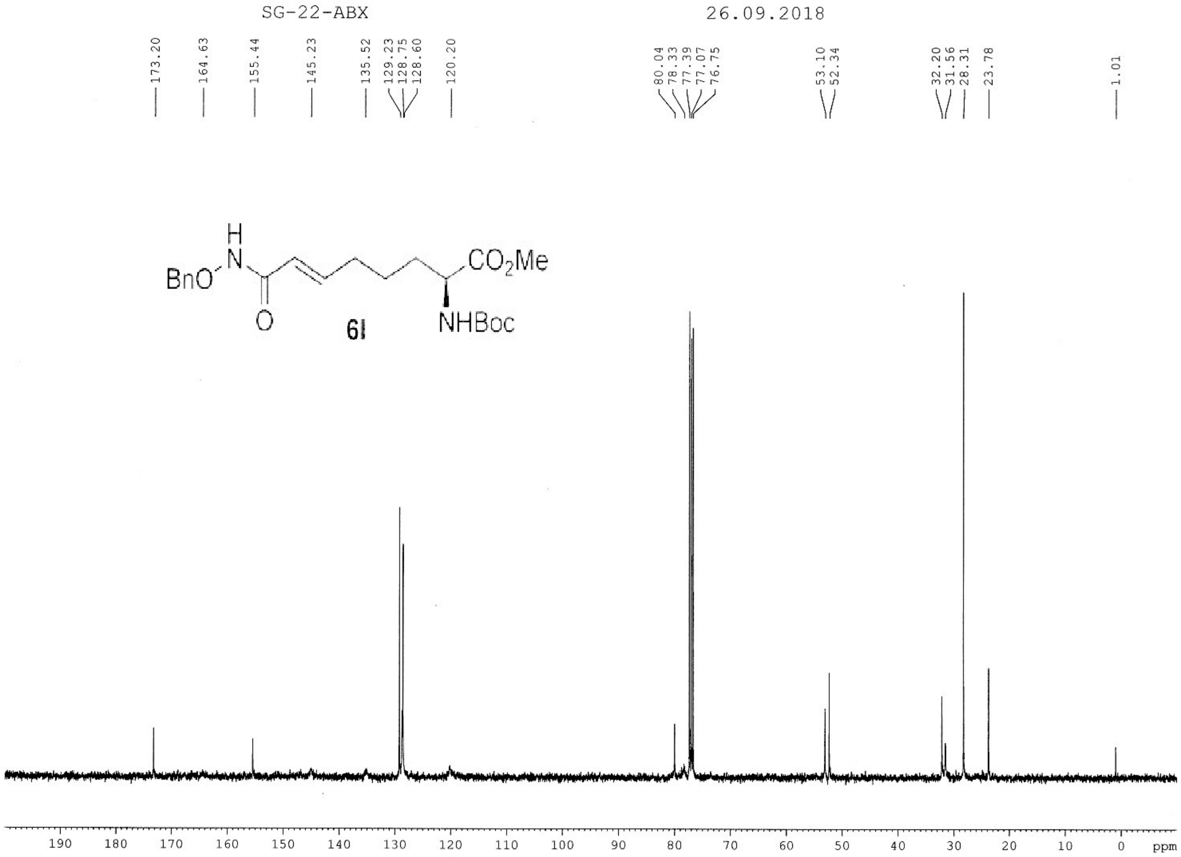

^{13}C NMR Spectrum of compound **6l** in DMSO-d_6

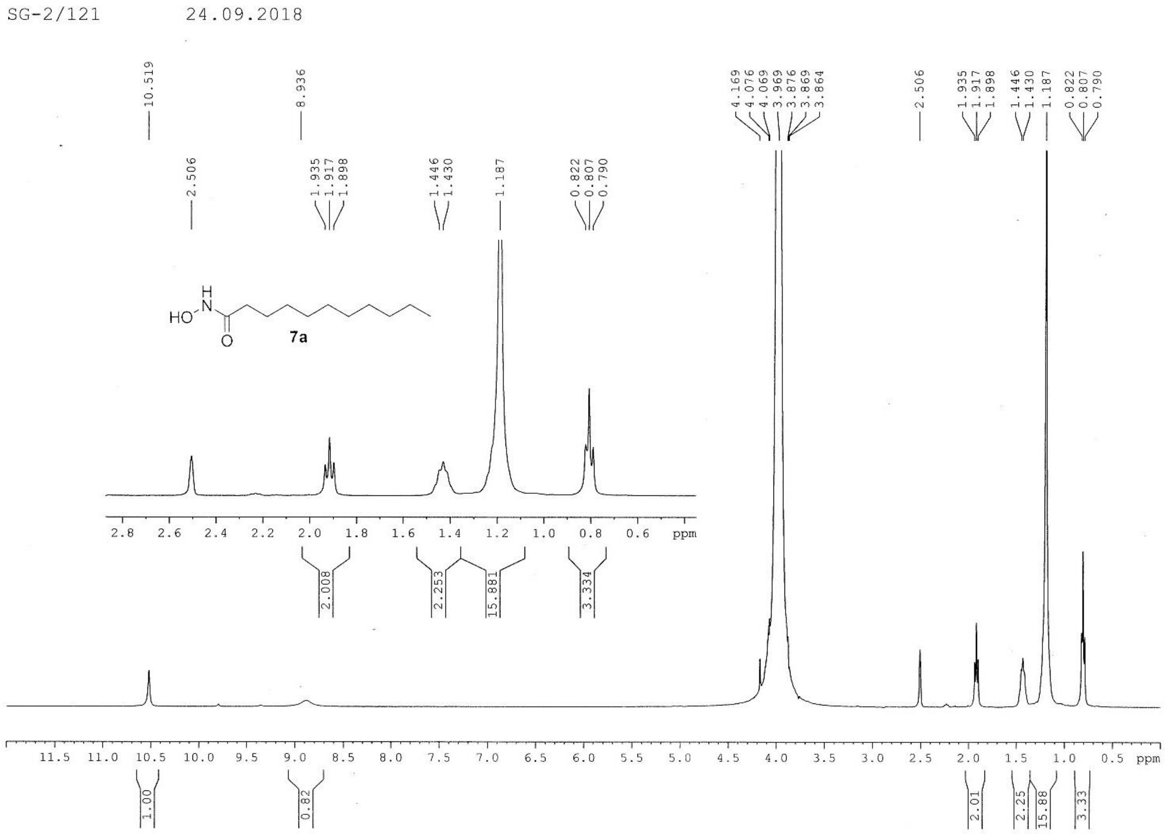

^{1}H NMR Spectrum of compound **7a** in DMSO-d_6

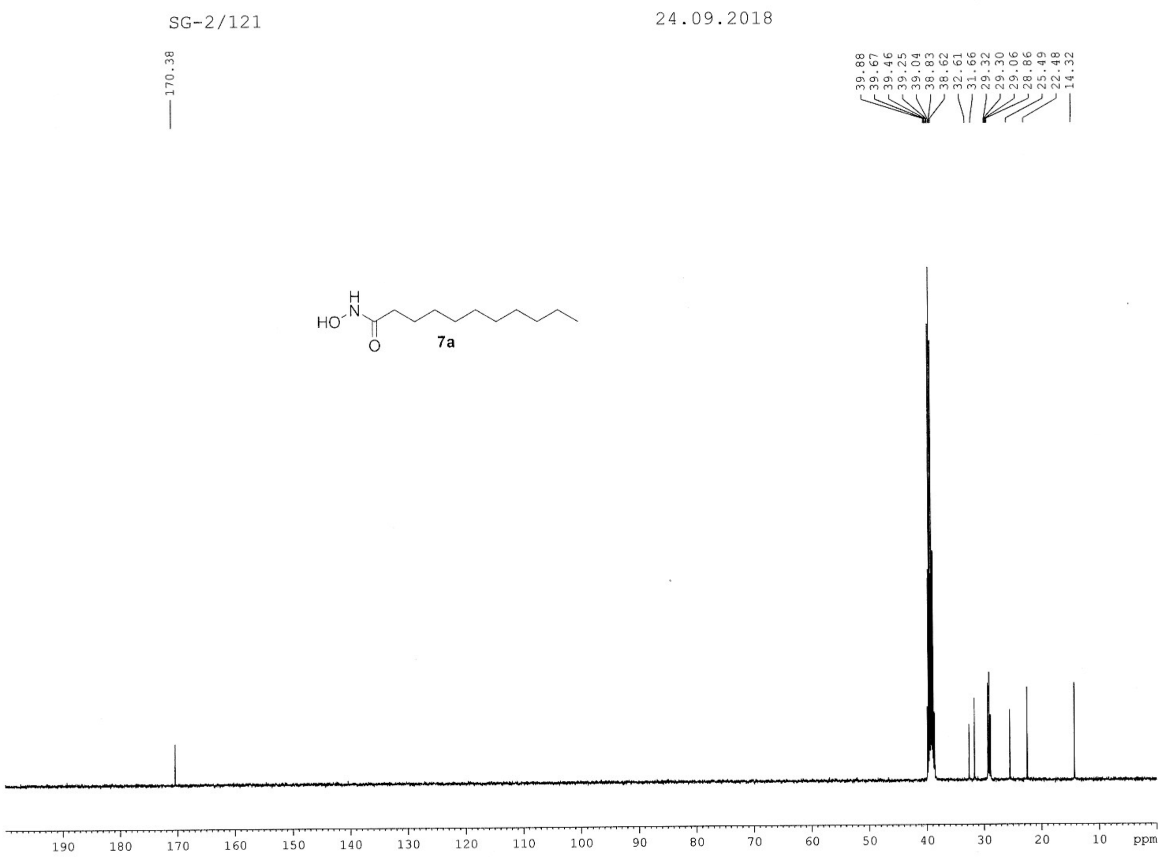

^{13}C NMR Spectrum of compound **7a** in DMSO-d_6

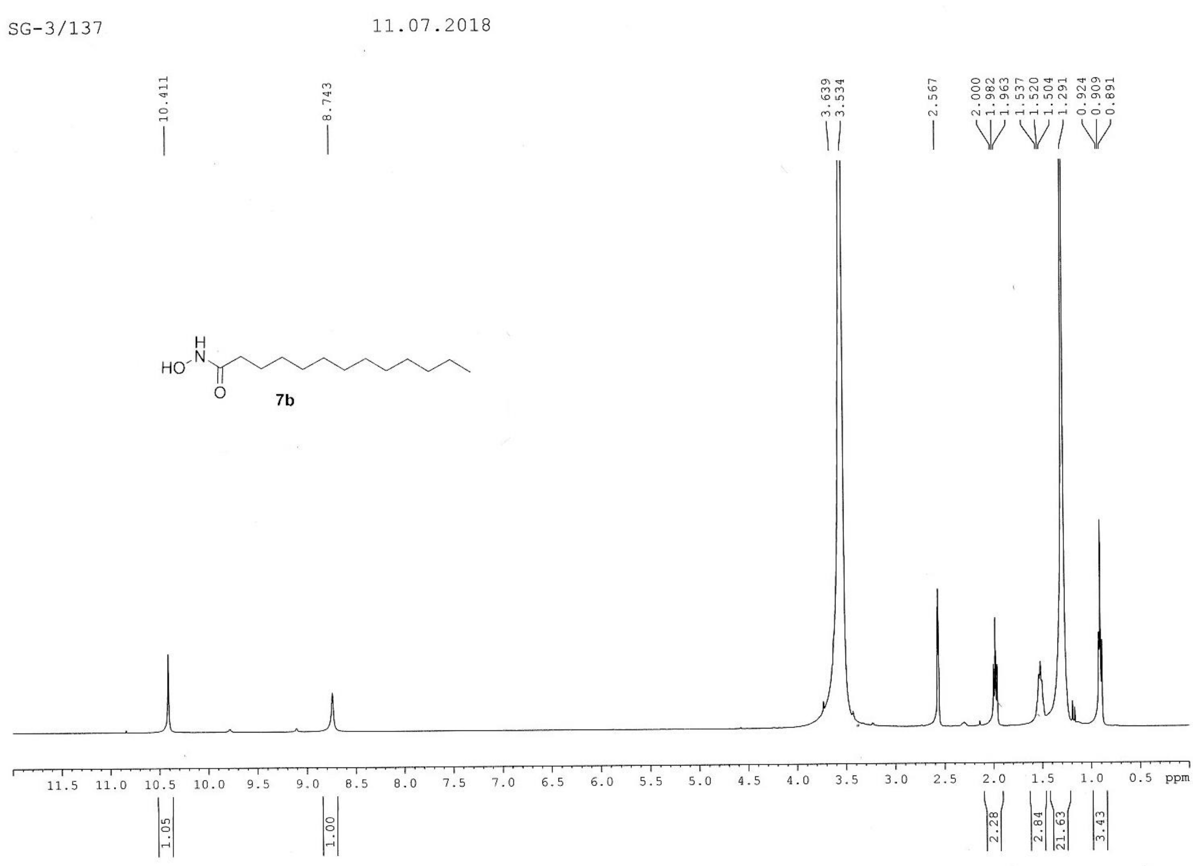

^{1}H NMR Spectrum of compound **7b** in DMSO-d_6

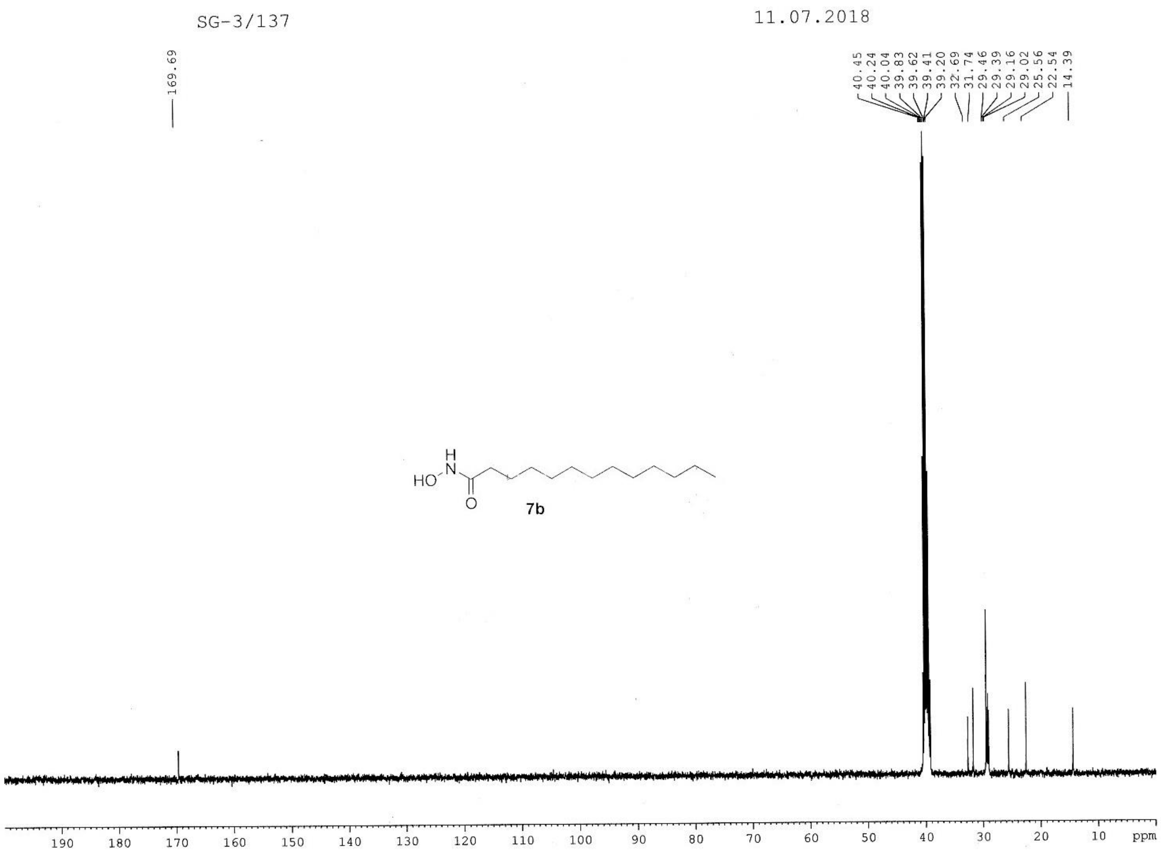

^{13}C NMR Spectrum of compound **7b** in DMSO-d_6

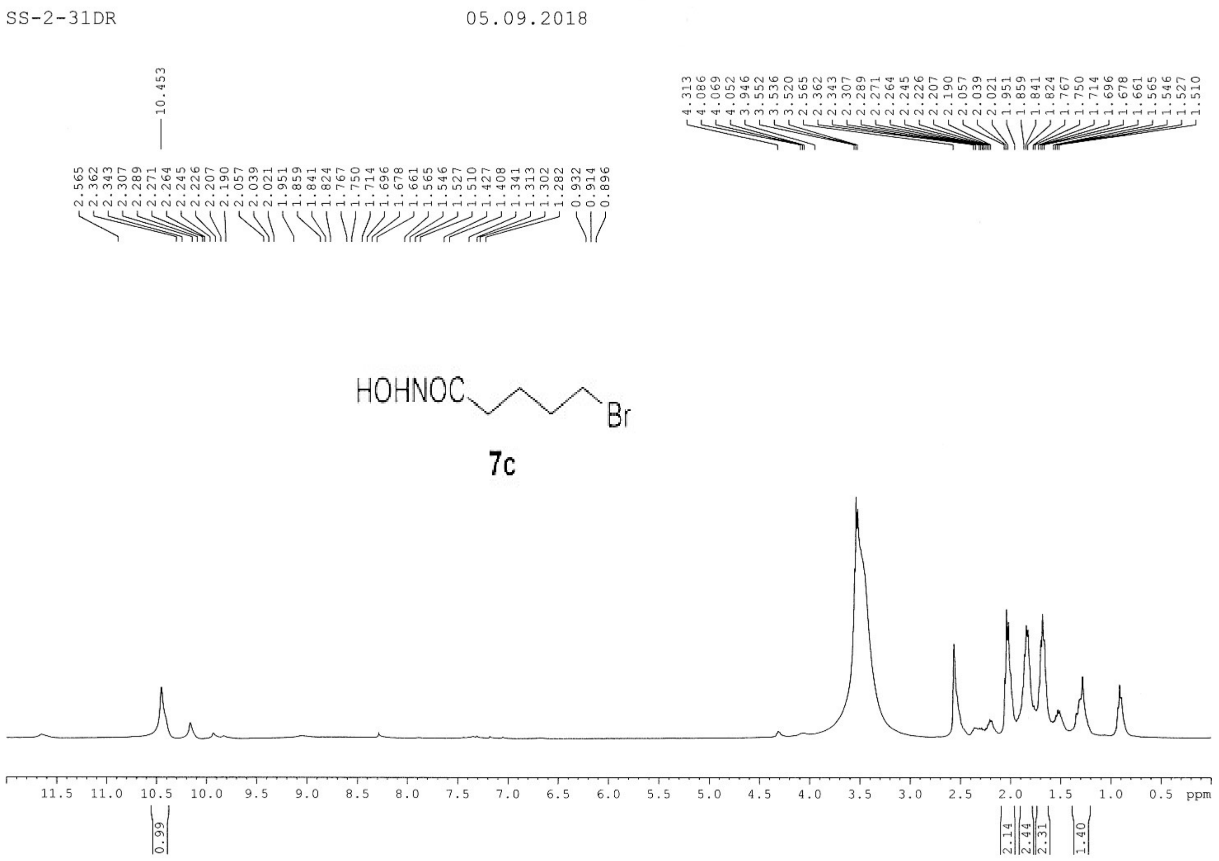

^{1}H NMR Spectrum of compound **7c** in DMSO-d_6

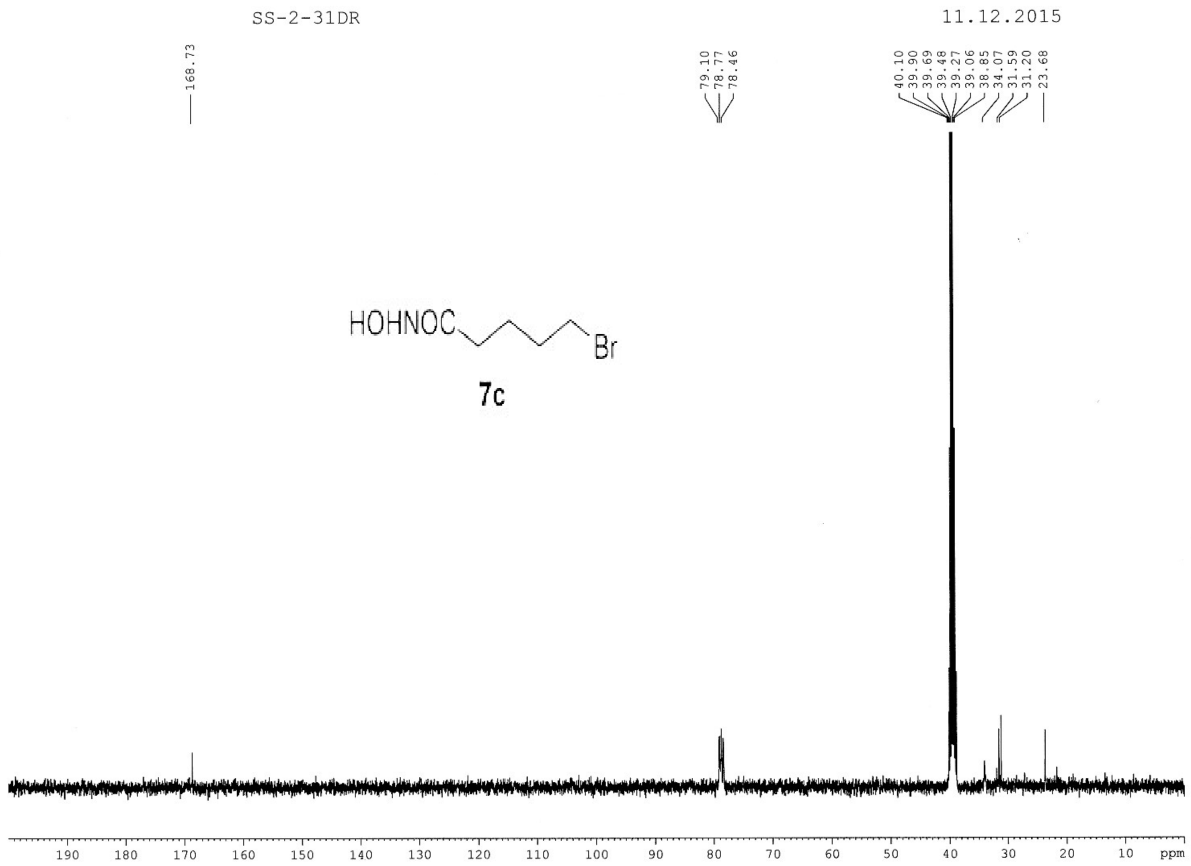

^{13}C NMR Spectrum of compound **7c** in DMSO-d_6

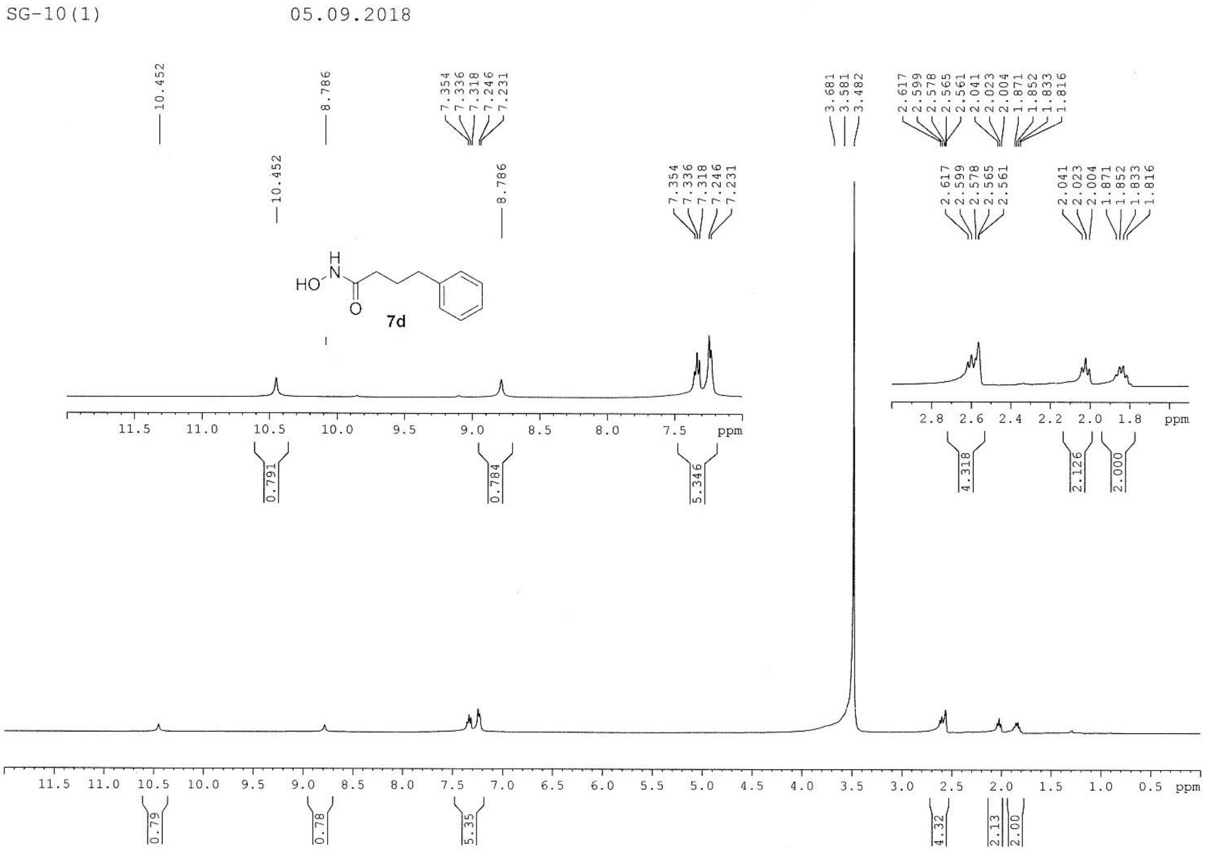

^{1}H NMR Spectrum of compound **7d** in DMSO-d_6

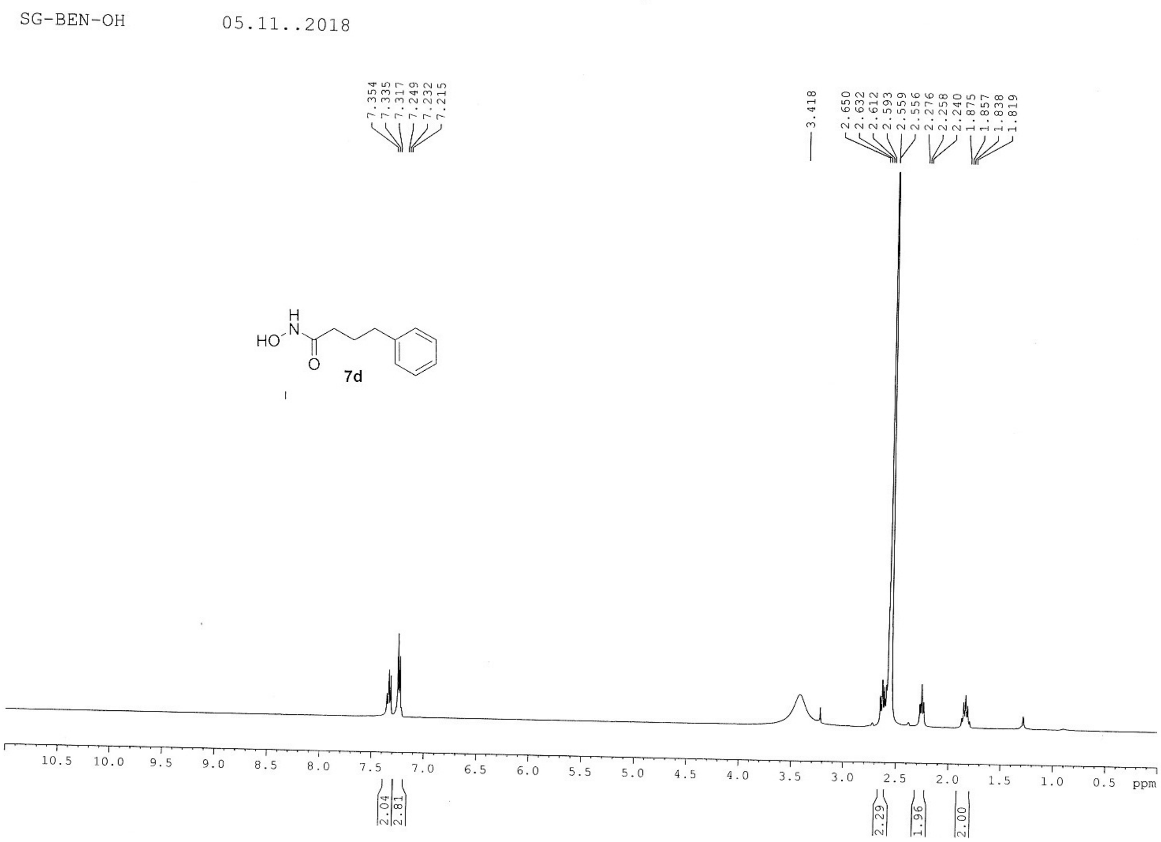

^{1}H NMR Spectrum of compound **7d** in dry DMSO-d_6

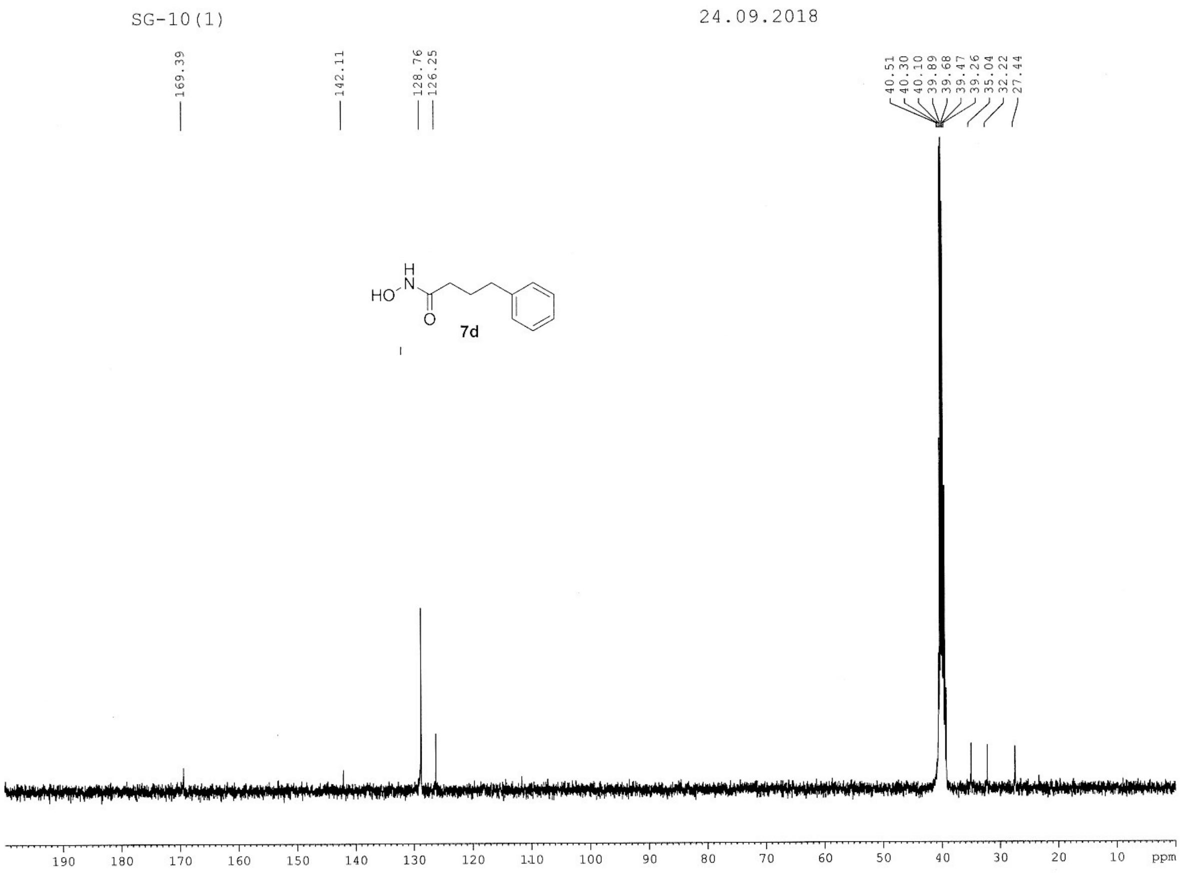

^{13}C NMR Spectrum of compound **7d** in DMSO-d_6

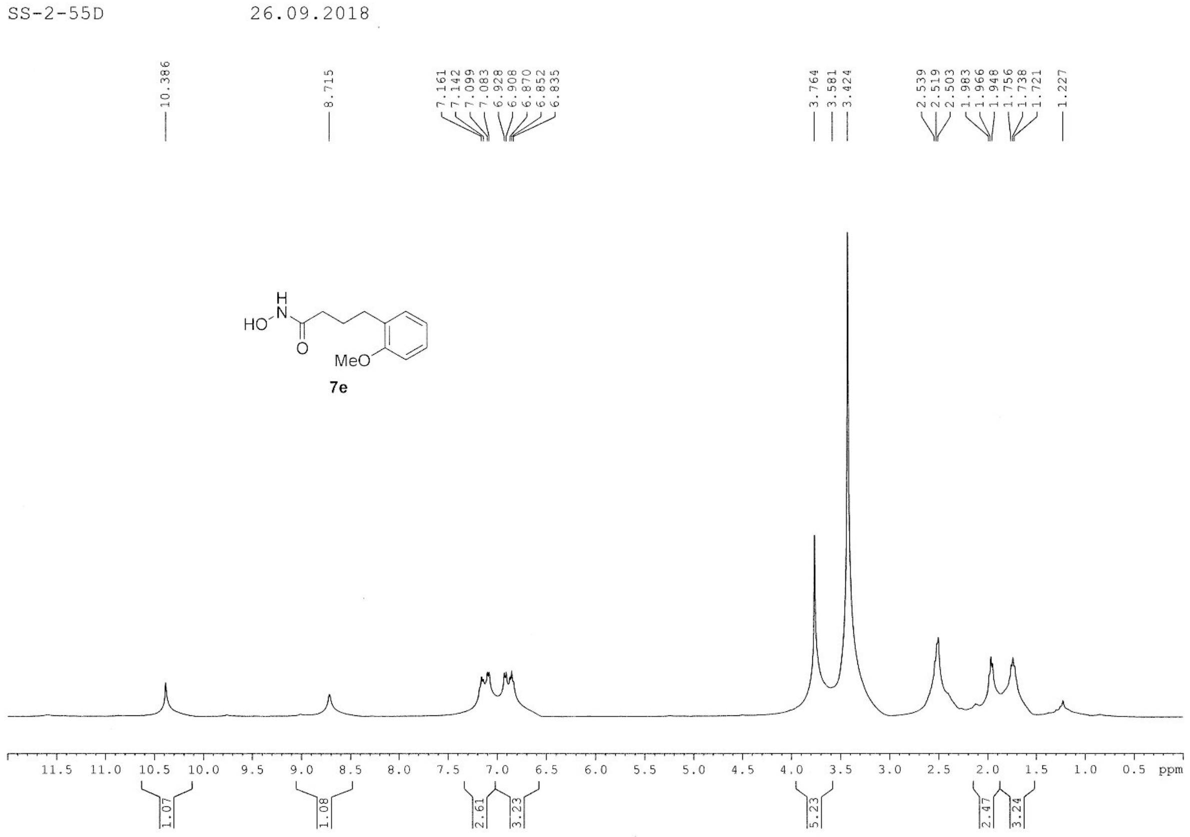

^{1}H NMR Spectrum of compound **7e** in DMSO-d_6

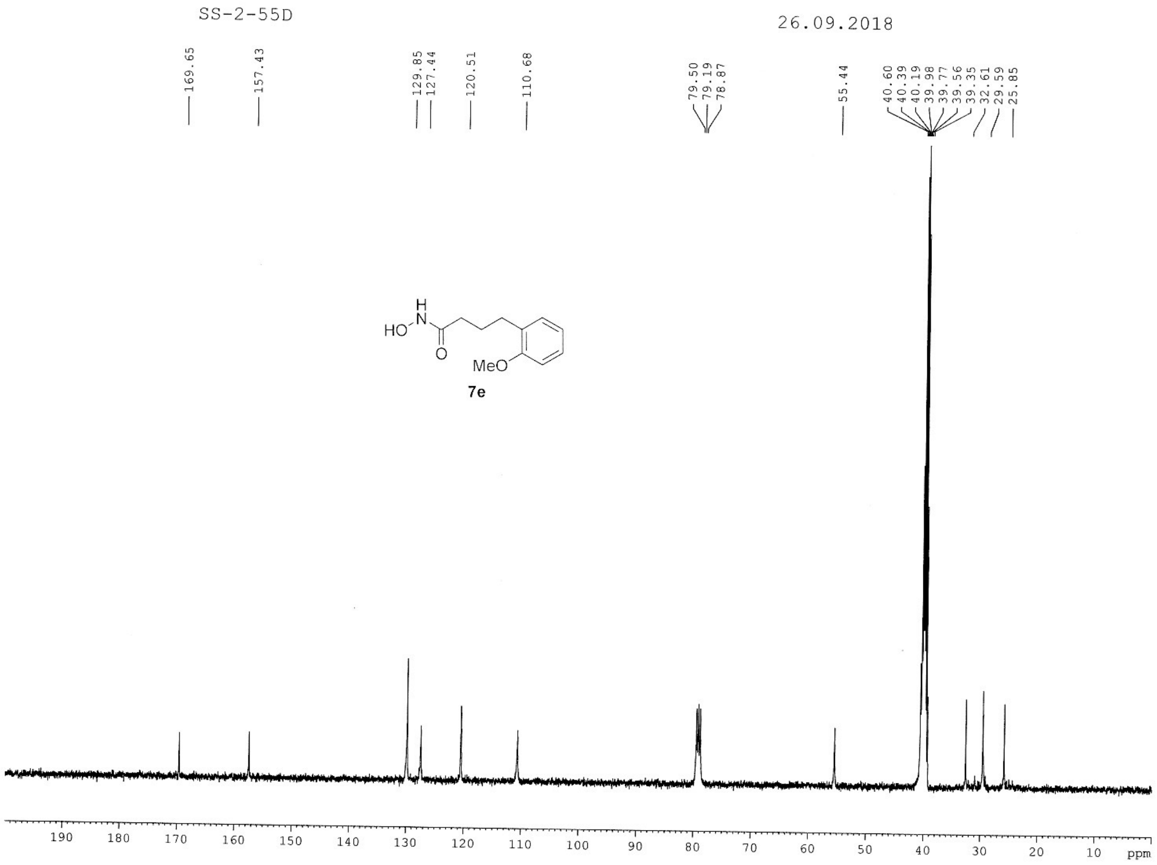

^{13}C NMR Spectrum of compound **7e** in DMSO-d_6

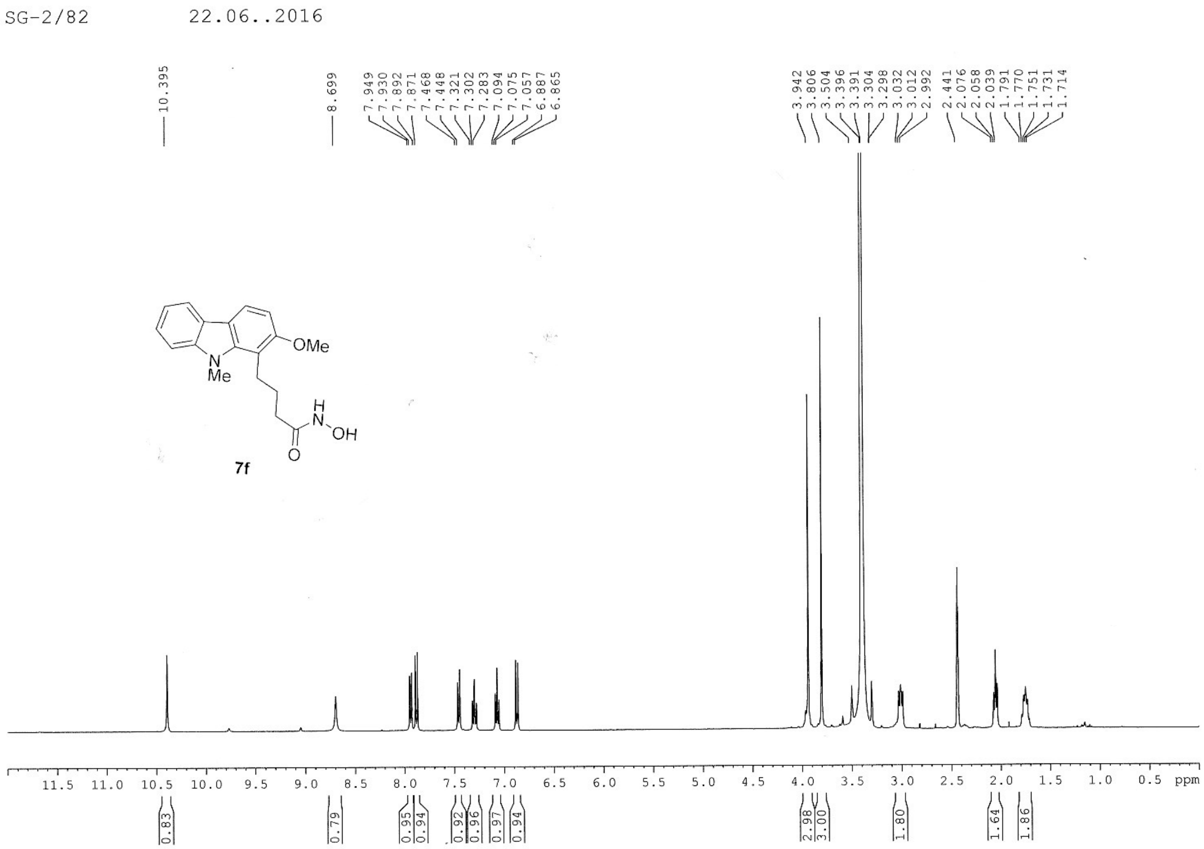

^{1}H NMR Spectrum of compound **7f** in DMSO-d_6

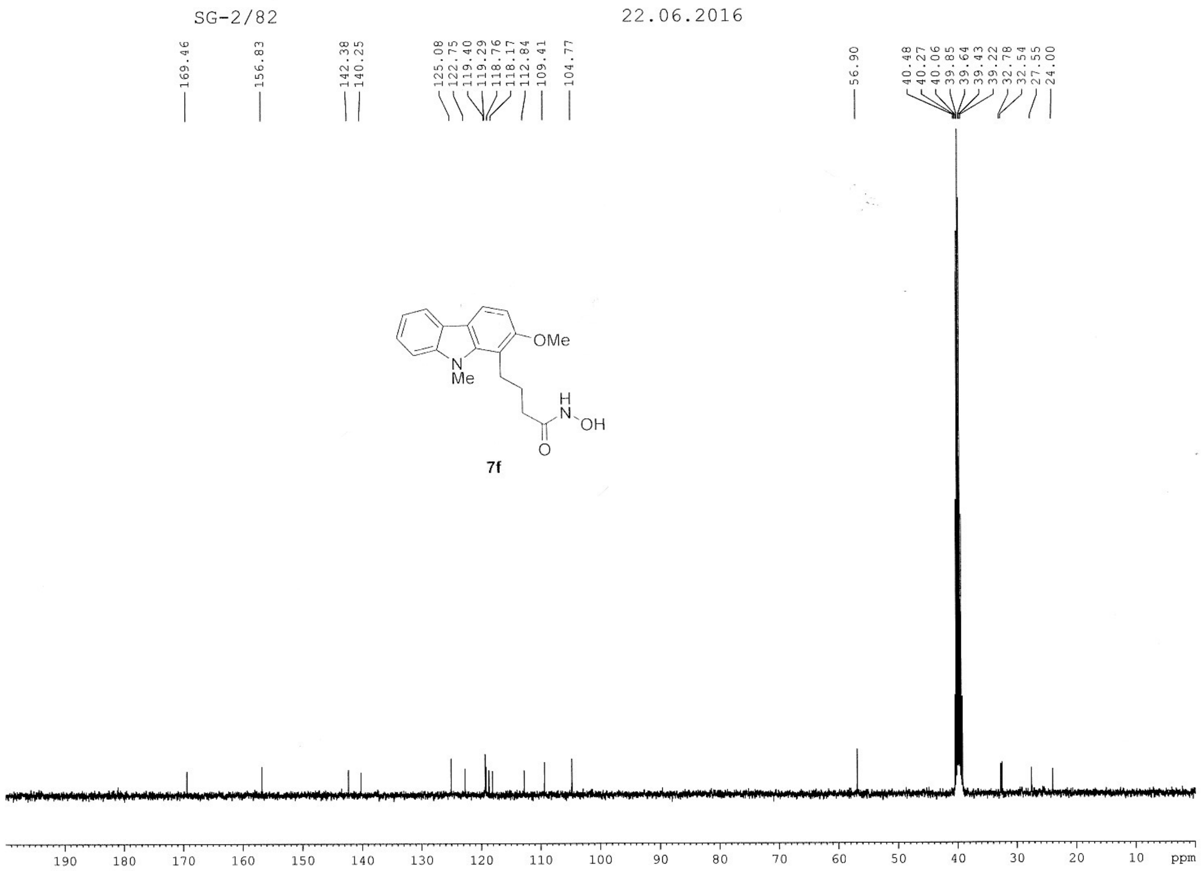

^{13}C NMR Spectrum of compound **7f** in DMSO-d_6

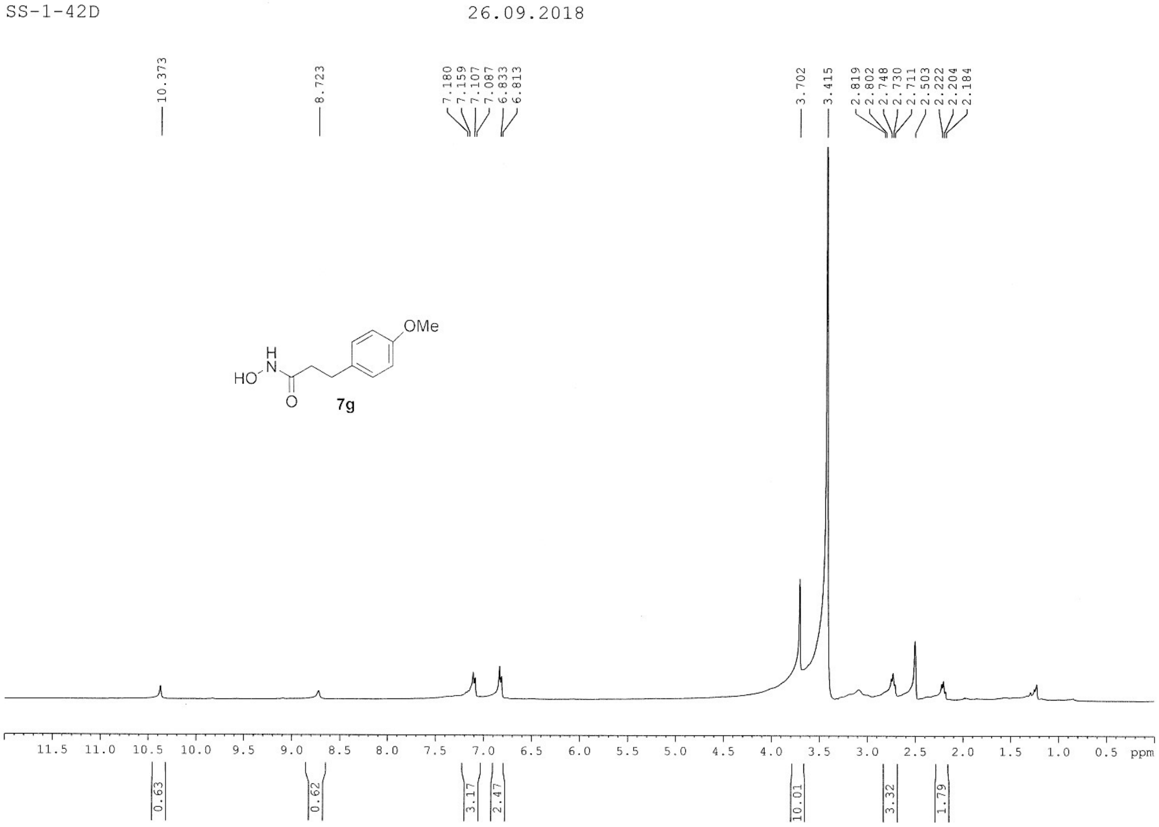

^{1}H NMR Spectrum of compound **7g** in DMSO-d_6

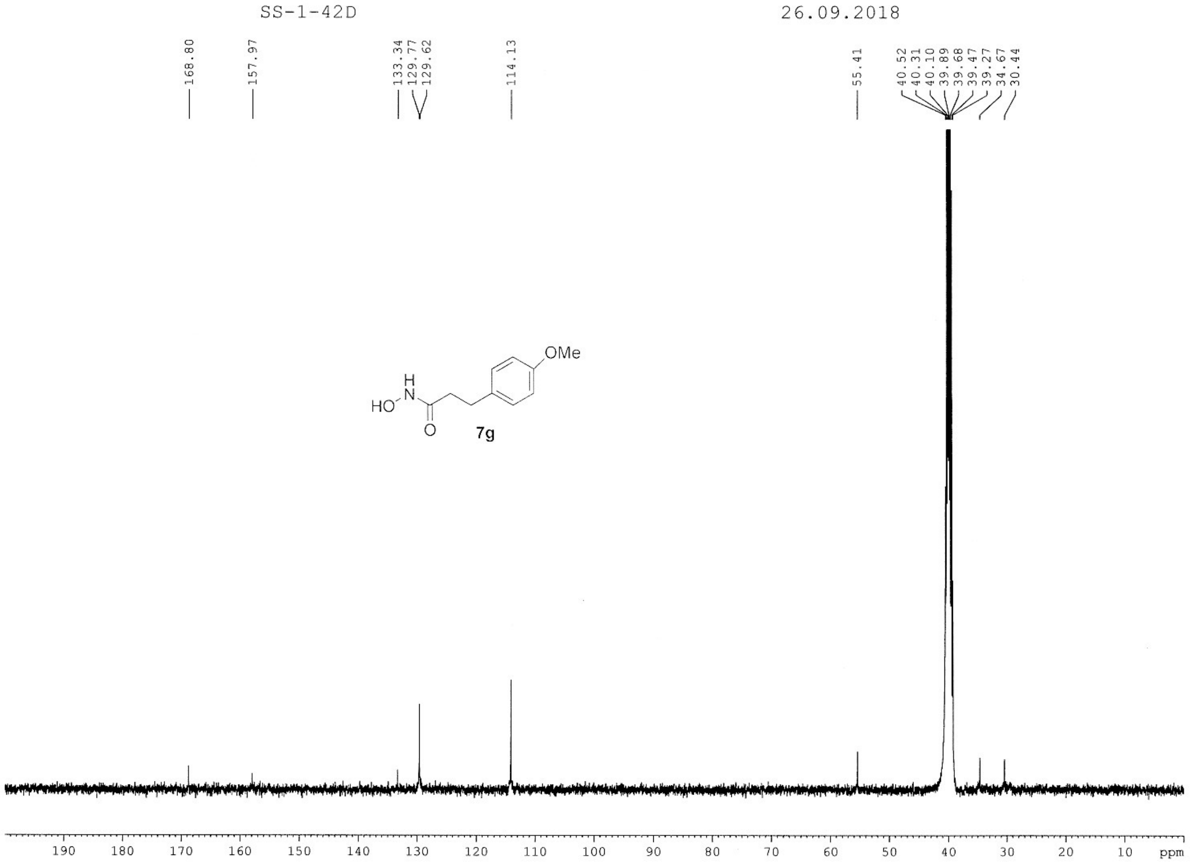

^{13}C NMR Spectrum of compound **7g** in DMSO-d_6

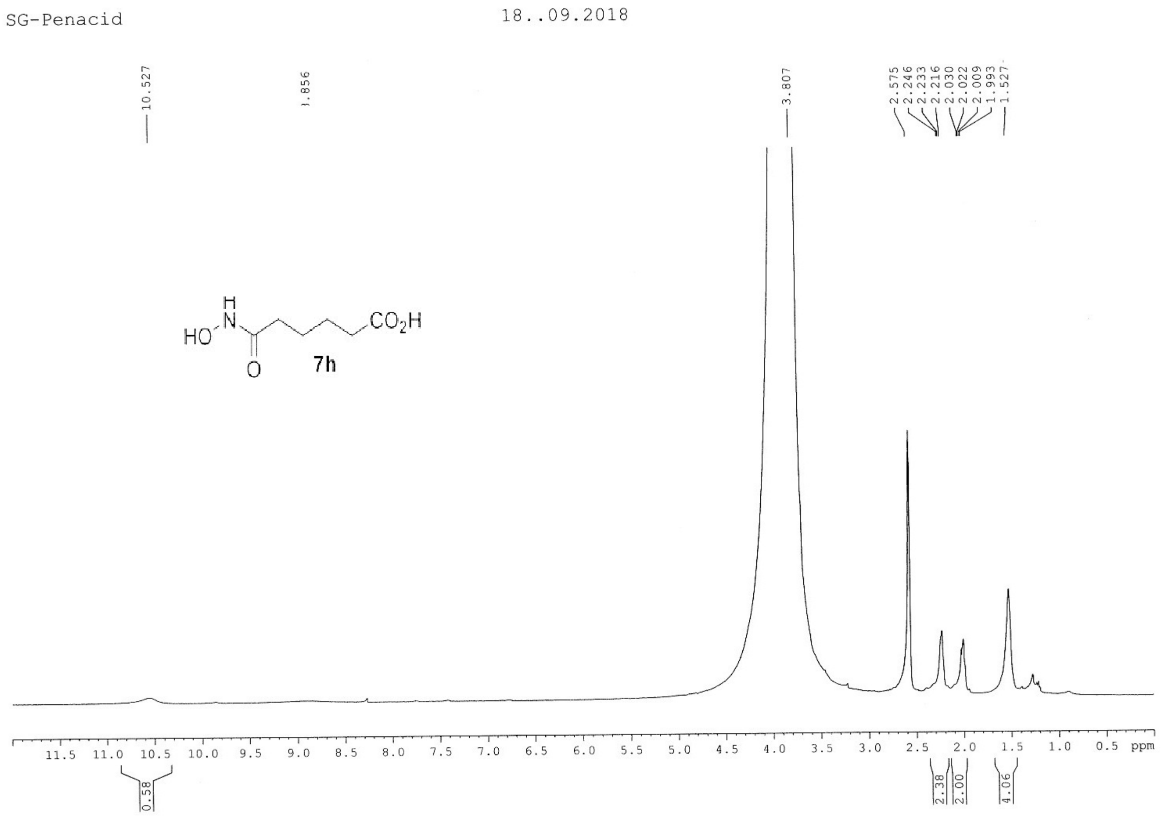

^{1}H NMR Spectrum of compound **7h** in DMSO-d_6

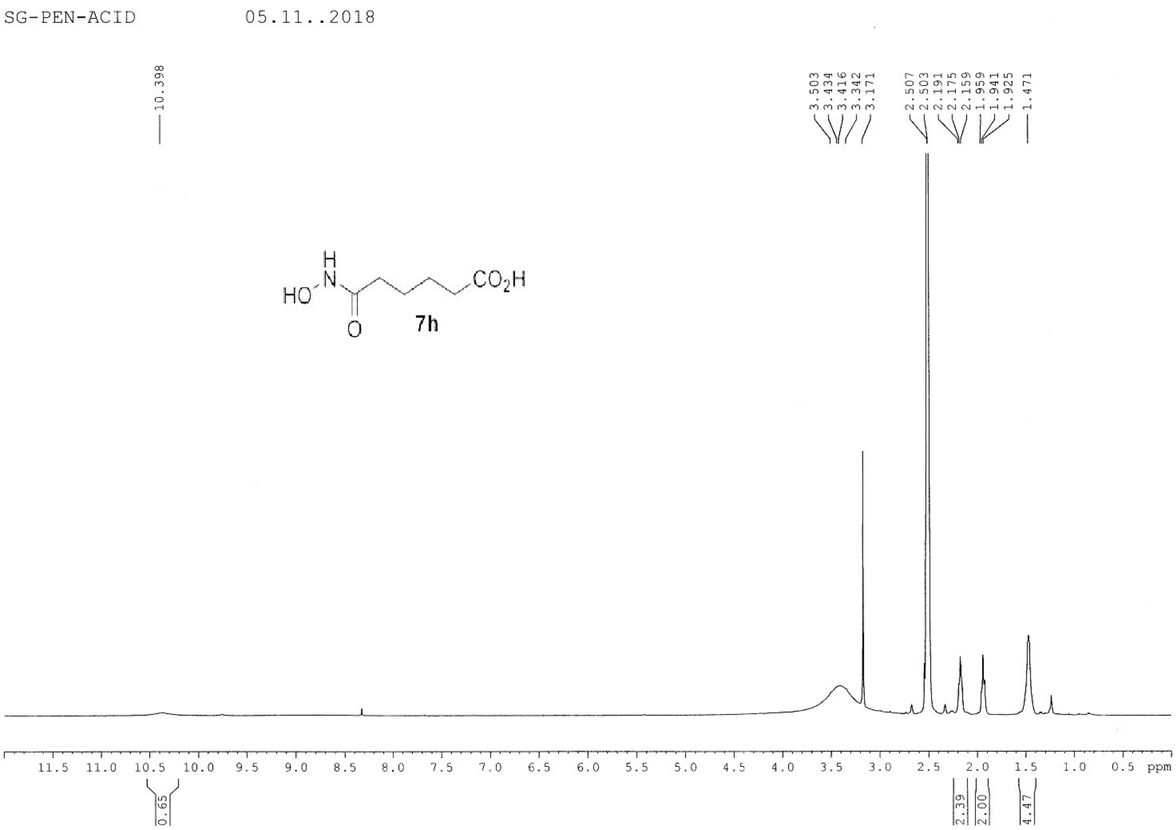

^{1}H NMR Spectrum of compound **7h** in dry DMSO-d_6

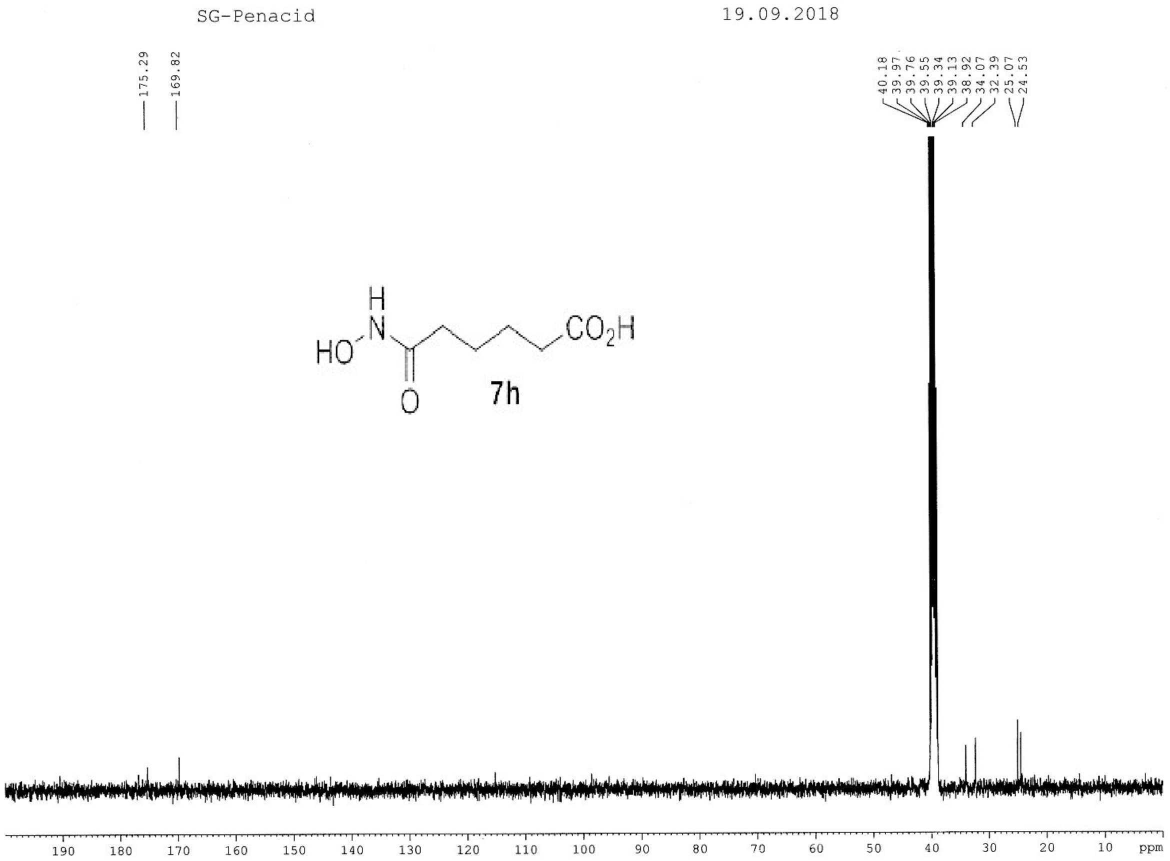

^{13}C NMR Spectrum of compound **7h** in DMSO-d_6

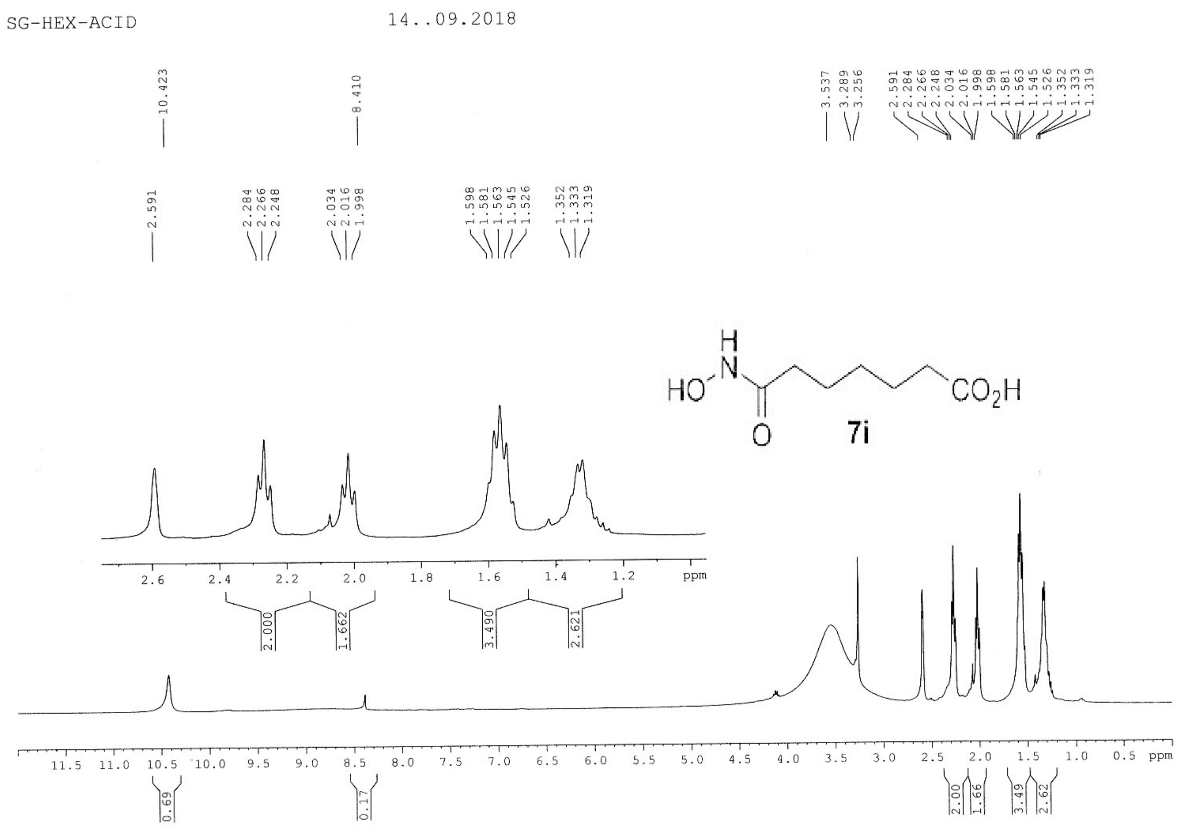

^{1}H NMR Spectrum of compound **7i** in DMSO-d_6

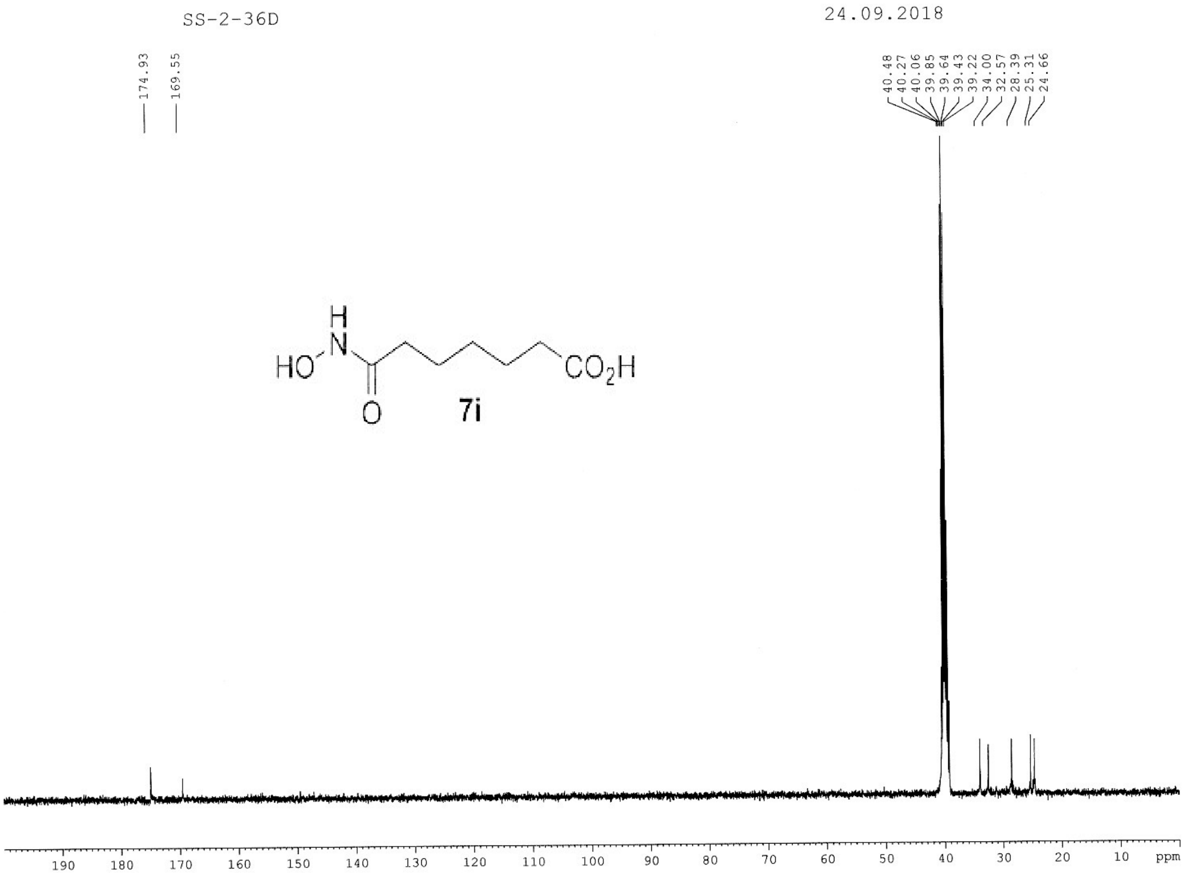

^{13}C NMR Spectrum of compound **7i** in DMSO-d_6

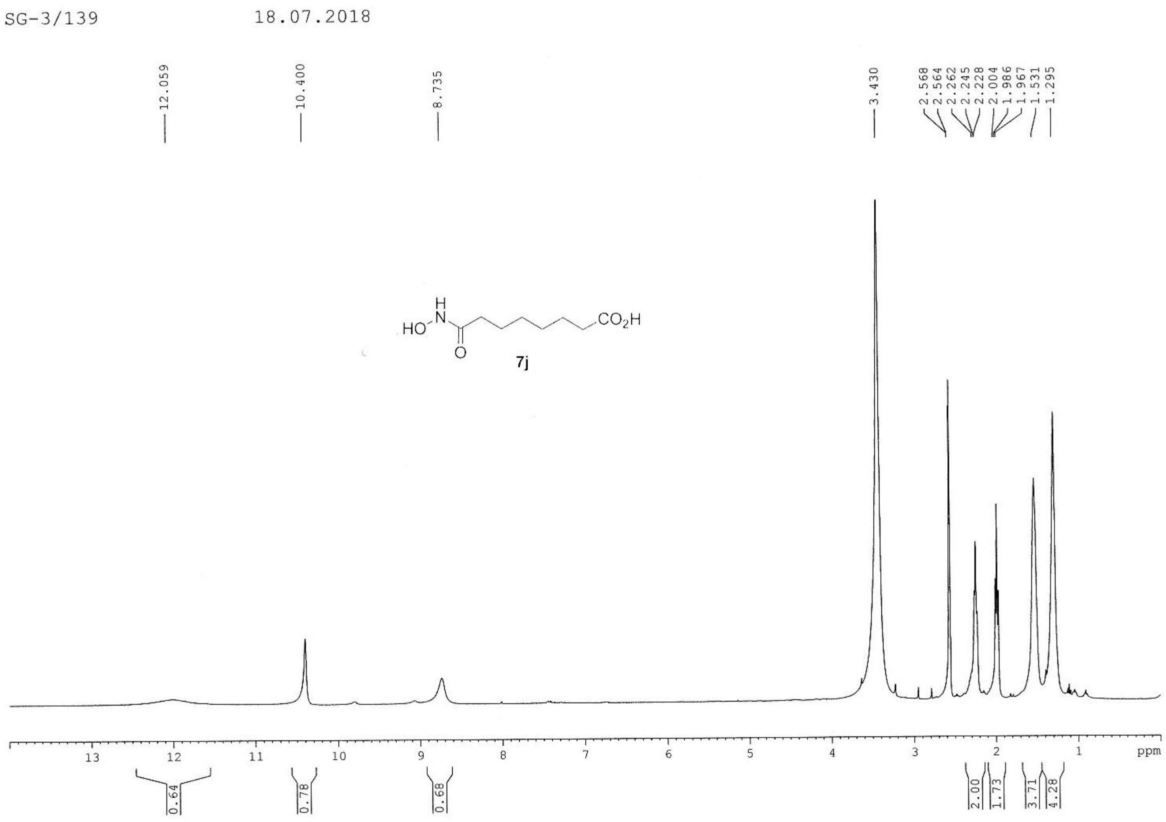

^{1}H NMR Spectrum of compound **7j** in DMSO-d_6

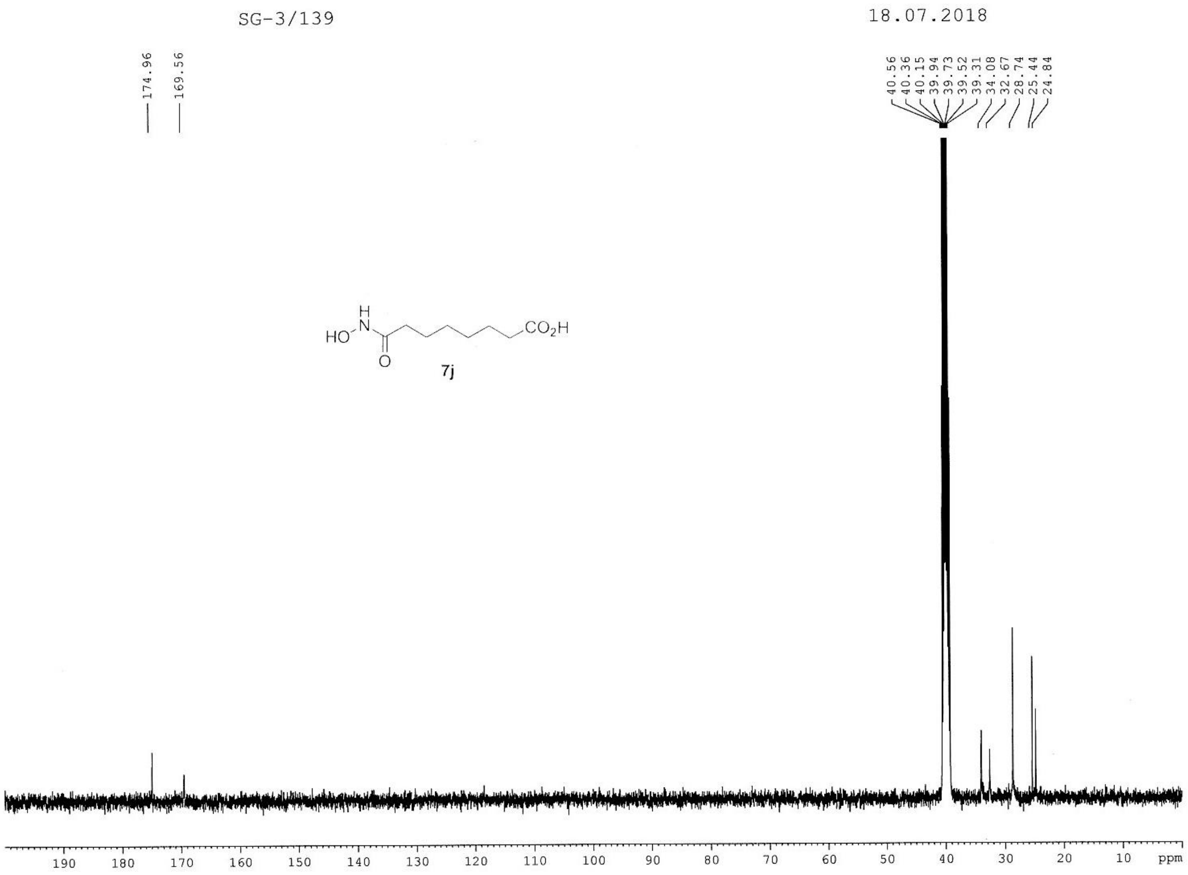

^{13}C NMR Spectrum of compound **7j** in DMSO-d_6

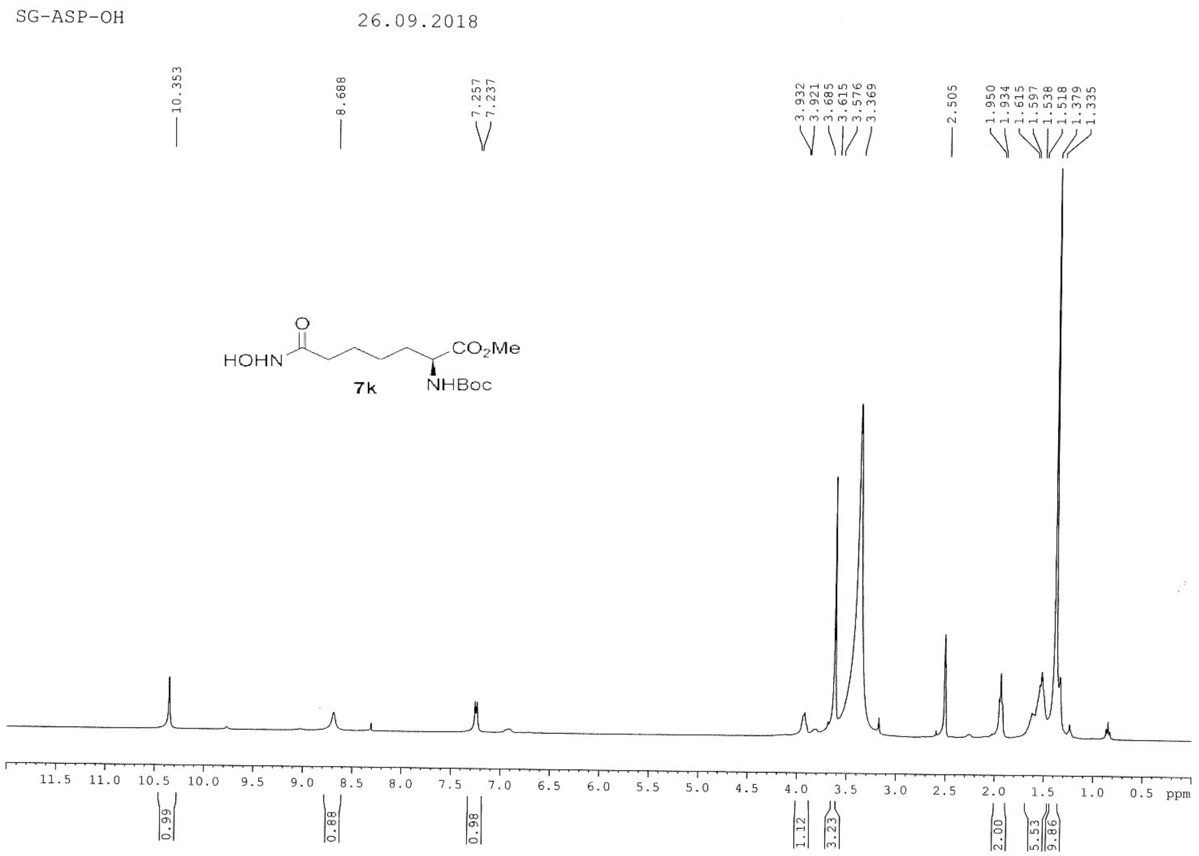

^{1}H NMR Spectrum of compound **7k** in DMSO-d_6

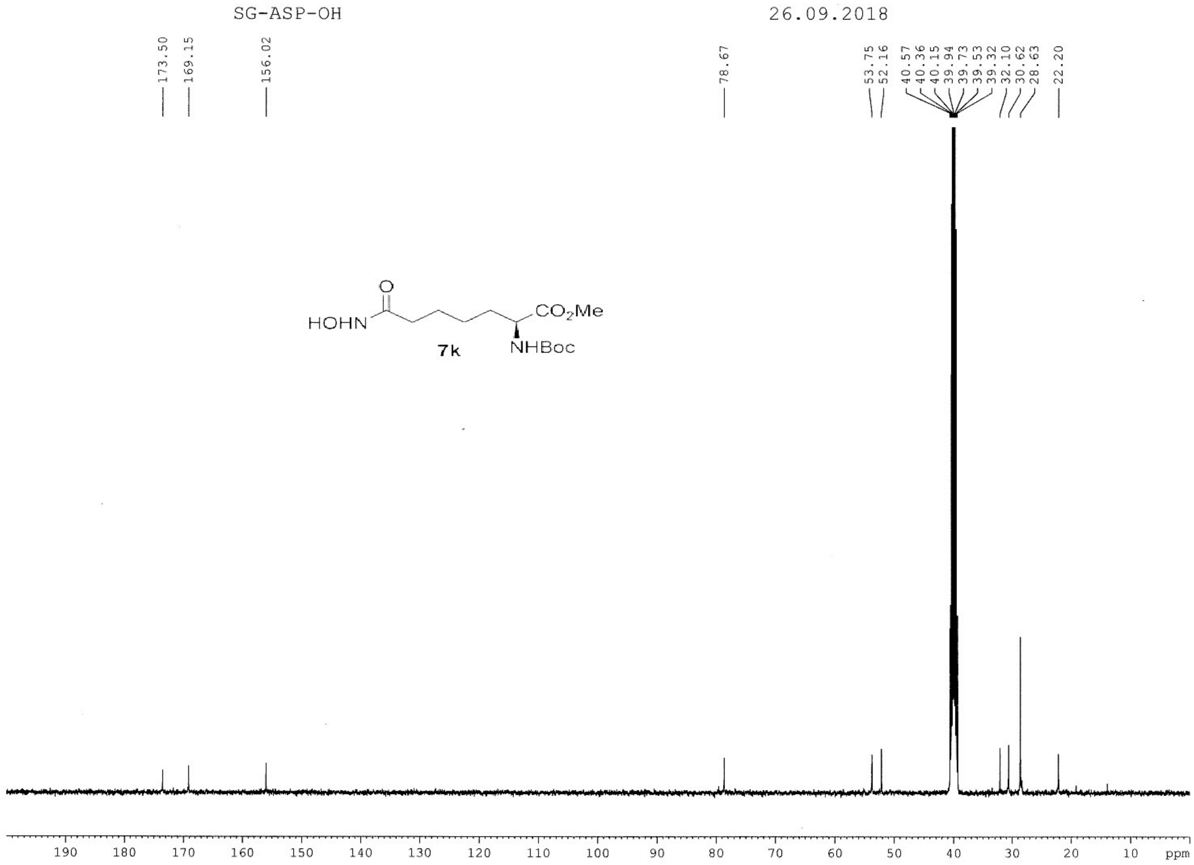

^{13}C NMR Spectrum of compound **7k** in DMSO-d_6

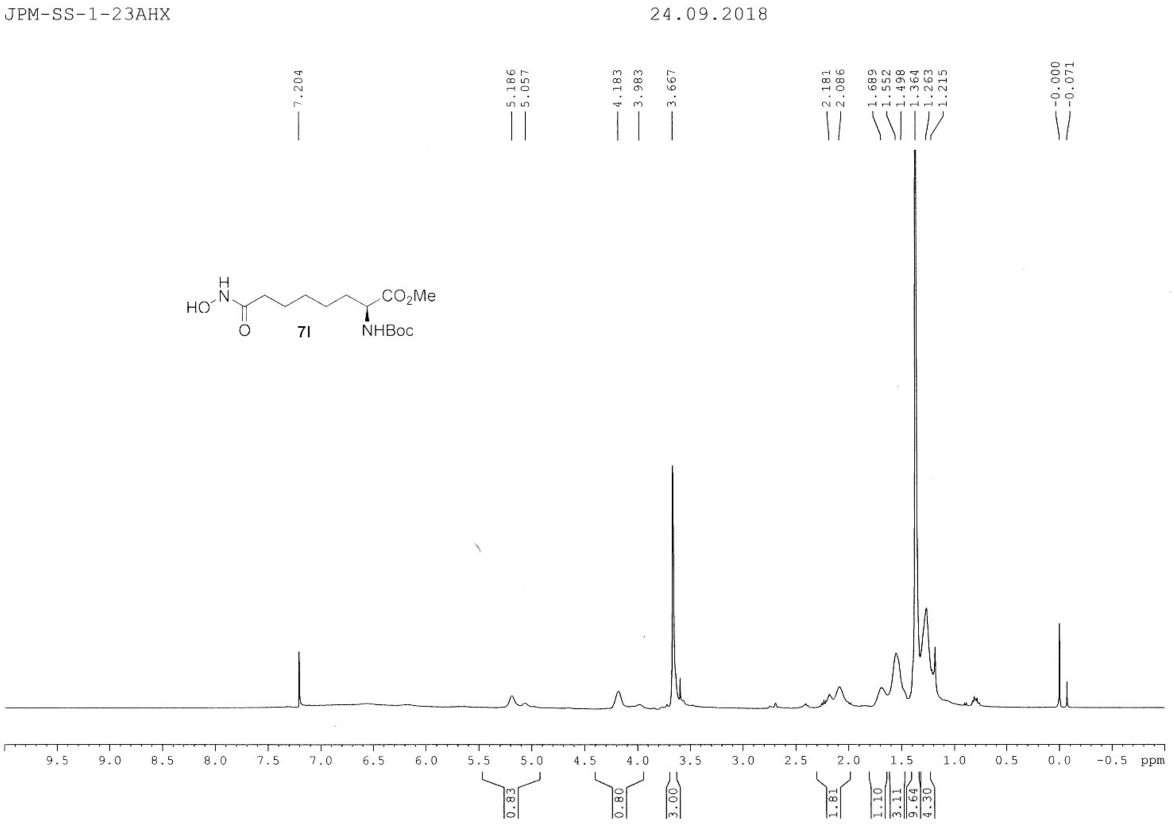

^{1}H NMR Spectrum of compound **7l** in $CDCl_3$

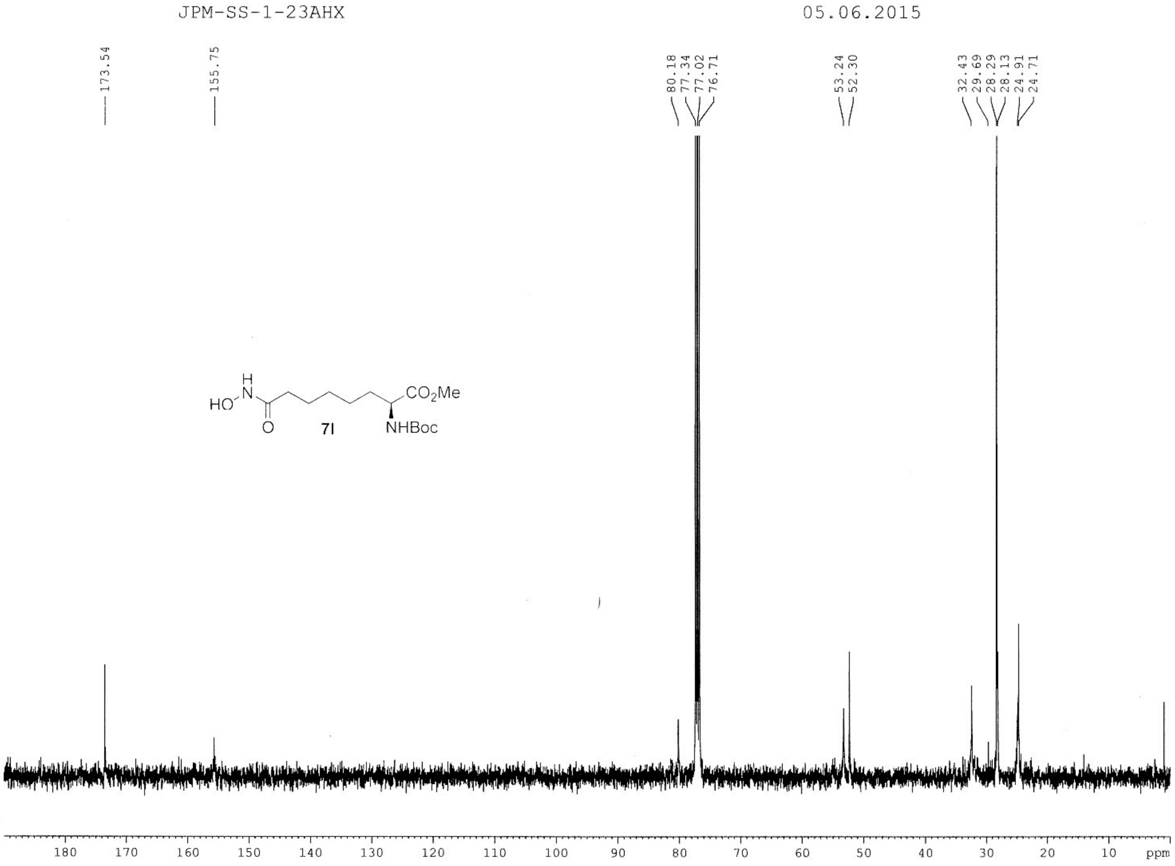

^{13}C NMR Spectrum of compound **7l** in $CDCl_3$
